# Chromosome duplication causes premature aging via defects in ribosome quality control

**DOI:** 10.1371/journal.pbio.3003509

**Published:** 2025-11-17

**Authors:** Leah E. Escalante, James Hose, Jamie M. Ahrens, Hollis Howe, Norah Paulsen, Sofia J. Liss, Michael Place, Audrey P. Gasch

**Affiliations:** 1 Center for Genomic Science Innovation, University of Wisconsin-Madison, Madison, Wisconsin, United States of America; 2 Great Lakes Bioenergy Research Center, University of Wisconsin-Madison, Madison, Wisconsin, United States of America; 3 Department of Medical Genetics, School of Medicine and Public Health, University of Wisconsin-Madison, Madison, Wisconsin, United States of America; Yale University, UNITED STATES OF AMERICA

## Abstract

Down syndrome, caused by an extra copy of Chromosome 21, causes lifelong problems. One of the most common phenotypes among people with Down syndrome is premature aging, including early tissue decline, neurodegeneration, and shortened life span. Yet the reasons for premature systemic aging are a mystery and difficult to study in humans. Here we show that chromosome amplification in wild yeast also produces premature aging and shortens life span. Chromosome duplication disrupts nutrient-induced cell-cycle arrest, entry into quiescence, and cellular health during chronological aging, across genetic background and independent of which chromosome is amplified. Using a genomic screen, we discovered that these defects are due in part to aneuploidy-induced dysfunction in Ribosome Quality Control (RQC). We show that aneuploids entering quiescence display aberrant ribosome profiles, accumulate RQC intermediates, and harbor an increased load of protein aggregates compared to euploid cells. Although they maintain proteasome activity, aneuploids also show signs of ubiquitin dysregulation and sequestration into foci. Remarkably, inducing ribosome stalling in euploids produces similar aging phenotypes, while up-regulating limiting RQC subunits or poly-ubiquitin alleviates many of the aneuploid defects. We propose that the increased translational load caused by having too many mRNAs accelerates a decline in translational fidelity, contributing to premature aging.

## Abstract 2

Syndromes caused by chromosome amplification, such as Down syndrome, are characterized by premature aging, but the reason behind this is unclear. This study shows that chromosome amplification in yeast disrupts quiescence and lifespan due to defects in the ribosome quality control pathway, leading to protein aggregation and accelerated aging.

## Introduction

Chromosome amplification, here referred to as aneuploidy, is very detrimental during mammalian development and a leading cause of infertility in humans, for reasons that remain incompletely understood. Trisomy of human Chromosome 21 that causes Down syndrome (DS) is the only autosomal aneuploidy viable into adulthood, thanks to decades of medical advances to improve health [1,2]. However, one of the most penetrant hallmarks of DS and a remaining medical concern is premature aging, including premature skin wrinkling and hair loss, defects in tissue regeneration, and early-onset neurodegeneration including Alzheimer’s [3–6]. The reasons for premature systemic aging are largely a mystery, in part because the underlying cellular consequences of chromosome amplification remain unknown despite over 65 years of study.

Budding yeast *Saccharomyces cerevisiae* has been an excellent model to understand the cellular stress of chromosome amplification. Previous seminal work studying an aneuploidy-sensitized laboratory strain revealed that chromosome duplication in this strain produces myriad defects in cell metabolism, stress response, and management of protein homeostasis known as proteostasis [7–12]. In contrast, wild isolates of yeast studied to date are much more tolerant of aneuploidy, with milder growth defects during logarithmic growth and little sign of proteostasis stress unless cells are further taxed [13–15]. The reason for the phenotypic differences is traced to RNA-binding protein Ssd1, which is functional in wild strains but hypomorphic in the sensitized W303 lab strain [13,16]. Ssd1 is involved in translational control and mRNA localization, and binds to several hundred transcripts encoding diverse functions [17–21]. Deletion of *SSD1* from wild strains renders cells highly sensitive to chromosome amplification, with cells showing many of the signatures of the sensitized lab strain including proteostasis stress [13,22]. Recent modeling work from our lab points to a defect in translational regulation in *ssd1Δ* aneuploids [22–24]. Furthermore, aneuploid yeast, especially aneuploid strains lacking *SSD1*, are sensitive to translational inhibitors, including nourseothricin (NTC) that binds the ribosome and disrupts translation elongation [13,25,26]. Our hypothesis is that wild *S. cerevisiae* isolates can handle the stress of chromosome duplication, in part through Ssd1-dependent mechanisms, but cells may be close to their buffering capacity under standard growth conditions [13].

While studying wild aneuploid strains, we made an important discovery: although these aneuploids proliferate with some growth delay during exponential growth, they have a major defect entering and maintaining quiescence induced by nutrient exhaustion. Quiescence is an important state conserved across taxa, in which cells exit the cell cycle upon specific cues but retain the ability to re-initiate proliferation at a later time [27–30]. Quiescence is necessary for proper development and critical for growth control, tissue homeostasis, and cellular longevity [28,31–33]. In fact, people with DS and animal DS models have defects maintaining quiescent stem cells, a deficiency that may underlie other phenotypes associated with premature aging [34–36]. Haploid yeast has served as an important model for understanding the quiescent state and defining key stages of the process [30,37].

Here we show that chromosome amplification in yeast disrupts quiescence and life span due in part to defects in the Ribosome Quality Control (RQC) pathway. This pathway detects, disassembles, and clears collided ribosomes and the incomplete nascent polypeptides associated with them [38–40]. Part of the clearance mechanism involves non-mRNA-templated addition of alanine and threonine residues called “CAT” tails to the nascent-peptide C-terminus by Rqc2 (NEMF in humans), followed by ubiquitination by the E3 ligase Ltn1 (mammalian Listerin) that triggers proteasomal degradation [38,41–43]. Failure to clear stalled products, in particular CATylated peptides that are prone to aggregation, is associated with toxic aggregates and proteostasis collapse [43–46]. Defects in translation and RQC are known to accumulate with age [47–49]. Furthermore, RQC dysfunction contributes to neurodegeneration in mammals and human disease models, and neurons are particularly sensitive to protein aggregation and RQC defects [50–56]. In this work, we present a model for how aneuploidy produces translational errors and consequential protein aggregation, which disrupts several processes to accelerate aging.

## Results

### Chromosomal duplication disrupts quiescence and life span

In the course of ongoing investigations, we discovered that haploid derivatives of wild oak-soil strain YPS1009 with different chromosome duplications showed abnormal arrest and regrowth after nutrient exhaustion from rich medium. To investigate systematically, we characterized each step along the progression to quiescence in a series of engineered YPS1009 aneuploid strains each with a full duplication of a different chromosome [57]. In this strain background, euploid cells shift to respiration as glucose is depleted from the media (day 1 of culturing), arrest as unbudded cells in G0 (day 1–2), silence their transcriptome (day 1–7), and become small and dense (day 4–7). All of these steps are important for healthy life span [58–60].

**Fig 1:**
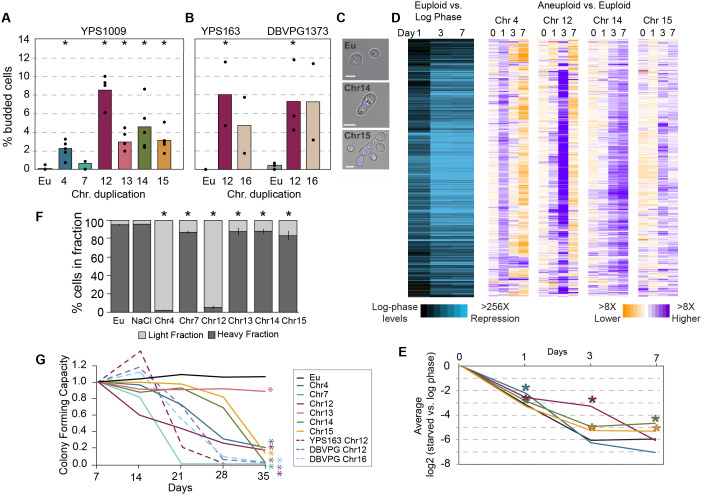
Chromosome duplication disrupts multiple signatures of quiescence. **(A, B)** Average and individual data points of percent budded cells is shown for visualization, at 2 days in (A) YPS1009 (*n* ≥ 3) or (B) oak strain YPS163 or vineyard strain DBVPG1373 (*n* = 2–3) with chromosome duplications as indicated. Asterisk, *p* < 0.05, Fisher’s exact test comparing counts of aneuploids vs. matched euploid (Eu) that were budded vs. unbudded. **(C)** Representative brightfield images from A. Blue, DAPI staining. Scale bar, 5 µm. **(D)** Left: Replicate-averaged log_2_(fold change) expression of 963 genes (rows) repressed in euploid but significantly higher in abundance (FDR < 0.05) in all four aneuploids compared to euploid, in at least one time point (see Methods). Right: The replicate-averaged log_2_(fold difference) in normalized transcript abundance for genes shown on the left, in each aneuploid vs. euploid at that time point. Purple indicates a repression defect. Data can be found in S2 Data. **(E)** Average of all genes shown in D for strains in the key to the left. Asterisk, *p* < 1 × 10^−8^, *T* test comparing the set of transcript abundances in each aneuploid vs. euploid at each time point. **(F)** Proportion of dense and light cells after 4 days (*n* = 3–5). Asterisk, *p* < 0.05, Chi-squared test. **(G)** Average fraction of colony-forming units relative to day 7 (YPS1009 strains, *n* = 3–6; YPS163 and DBVPG1373, *n* = 2). Asterisk, *p* < 0.05, replicate-paired *T* test comparing aneuploids to euploid at day 35. Euploid YPS163 and DBVPG1373 viability are indistinguishable from YPS1009. The data underlying this figure can be found in S2 Table.

We found that all aneuploids tested had defects in these quiescence hallmarks, to varying degrees. One of the earliest steps is cell-cycle arrest upon nutrient exhaustion in a saturated culture. Whereas nearly 100% of euploid cells arrest as unbudded cells by 2 days of culturing, conservatively 1%–12% of cells, depending on the amplified chromosome, showed morphology indicative of budding (Fig 1A), even though the cultures had completely exhausted glucose (S1A Fig). The arrest defect is not specific to YPS1009, as two other strain backgrounds with extra chromosomes showed similar defects (Fig 1B). Supplementation with extra glucose, amino acids, or yeast extract did not correct the issue. In addition to the arrest defect, YPS1009 with an extra chromosome XIV (YPS1009_Chr14) showed an unusual morphology of bi-lobed cells with a single round nucleus in between, while YPS1009_Chr15 took on a multi-budded elongated state (Fig 1C). Importantly, YPS1009_Chr14 and _Chr15 aneuploids do not show these morphologies during log-phase growth (S1B Fig), indicating that these morphologies are specific to late-culture growth when cells normally arrest. Thus, chromosome duplication generally disrupts cell-cycle arrest upon nutrient exhaustion from rich medium, albeit with heterogeneity and some chromosome-specific effects.

Aneuploid cultures also displayed defects in subsequent steps of quiescence, including the stereotypical transcriptome silencing associated with quiescence [59,61,62]. Euploid cultures repressed thousands of transcripts, >256-fold below log-phase levels, beginning 1 day past exponential phase and dropping to stable levels by day 3 (Fig 1D and 1E). We measured bulk transcriptomes of YPS1009 with a duplication of Chr4, Chr12, Chr14, or Chr15, normalized by cell number using spike-in of *Schizosaccharomyces pombe* cells (see Methods). The transcriptional profiles of all aneuploids were dysregulated to varying extents: we identified 963 transcripts that were reduced in euploid cells but at statistically significantly higher abundance in all four aneuploids, in at least one time point (false discovery rate [FDR] < 0.05, see Methods). Two of the aneuploids (YPS1009_Chr4 and Chr12) eventually reached euploid repression after a delay. Transcriptome silencing is known to be important for quiescence, since defective silencing shortens life span [63]. In addition to the silencing defect, aneuploidy also disrupted cell densification that occurs during this time frame. Unlike euploid cells that densify after 4 days of culturing, all aneuploids tested showed statistically significant effects, with YPS1009_Chr4 and _Chr12 showing the largest defect (Figs 1F and S1C). Cells treated with salt (NaCl) stress as a control showed no difference, showing that the effect is not explained by generalized stress sensitivity of the aneuploid cells.

Quiescence is fundamentally important for normal life span in yeast [31,32,64,65]—indeed, aneuploids have a substantially shorter chronological life span (Fig 1G). Euploids of multiple strain backgrounds exhibited near 100% colony-forming capacity over 5 weeks of culturing. Instead, all but one aneuploid tested exhibited a dramatically shorter life span, with few, if any, viable cells remaining by the end of the time course (Fig 1G). The lone exception, YPS1009_Chr13, whose viability was relatively stable over 5 weeks but started at a significantly lower levels (60%–70% of cells). The reduced life span was also seen in two other strain backgrounds, again indicating that the effect is not specific to YPS1009. Our results expand past studies showing that chromosome duplication in the aneuploidy-sensitized W303 lab strain shortens replicative life span [66]; however, interpretation of those results are confounded since the strain lacks functional Ssd1 that is linked to both life span and quiescence [20,60,67,68]. Our results show that even in the presence of *SSD1*, aneuploidy disrupts normal aging.

Collectively, our results show that chromosome amplification disrupts multiple signatures of quiescence and life span, independent of which chromosome is amplified, but with some chromosome-specific effects. This strongly suggests a generalizable consequence of chromosome duplication on aging and life span, overlayed with chromosome-specific impacts.

### A genetic screen for life span extension implicates the RQC pathway

**Fig 2:**
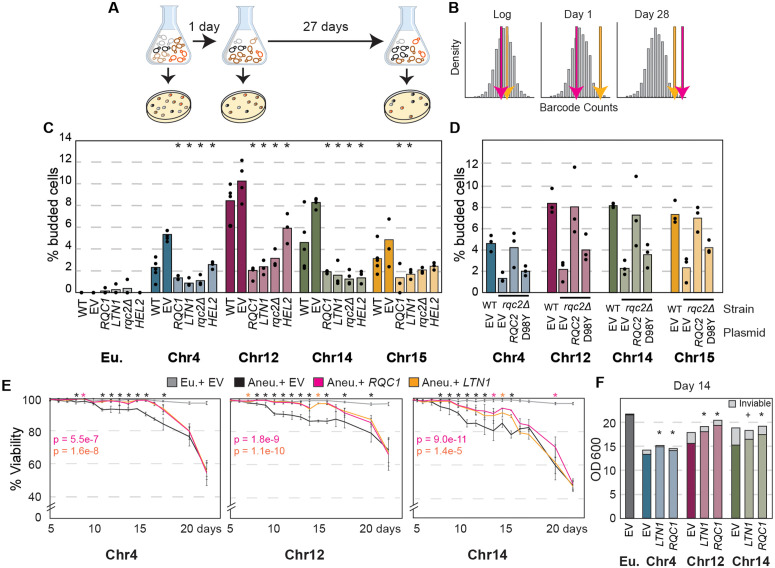
Genetic perturbation of the RQC pathway modulates cell-cycle defects. **(A)** Genetic screen approach, see text. **(B)** Representation of the density of all barcode counts in the library (gray) and an indicator for one gene enriched in the pool already at day 1 (orange) and a second gene enriched only at day 28 (pink). **(C)** Percent budded cells in euploid (Eu) and aneuploid cultures harboring empty vector (EV) or plasmids encoding *RQC1*, *LTN1*, or *HEL2* or in a strain lacking *RQC2* (*rqc2Δ*) after 2 days of culturing. Asterisk, *p* < 0.05 comparing strains with gene plasmids to EV or *rqc2Δ* to WT; *n* = 3, Fisher’s exact test applied to count data. (**D)** As in C but with plasmids expressing wild-type *RQC2* or the *rqc2 D98Y* mutant. None of the D98Y mutants were statistically different from the paired *rqc2∆* strain (*p* > 0.1, Fisher’s exact test). (**E)** Average and standard deviation of percent viability (**n** = 4 or as listed in S2 Table) over time in denoted strains. Listed p-values calculated using a two-way ANOVA comparing each strain across time to the EV control. Asterisks above each plot represent a one-tailed, replicate-paired *T* test assessing individual timepoints, colored according to the key. (**F)** Average optical density at day 4. * *p* < 0.05, + = 0.06, one-tailed *T* test compared to paired EV control. See S2 Table for count data and p-values. The data underlying this figure can be found in S2 Table.

To understand the mechanisms of life span limitation, we conducted a genetic screen in YPS1009_Chr12 cells as a representative (Fig 2A). We transformed the euploid and aneuploid strains with a low-copy, barcoded plasmid library expressing each of ~5,000 yeast genes flanked with their native upstream and downstream sequences [69]. YPS1009_euploid or YPS1009_Chr12 cells transformed with the library were pooled separately, grown to saturation, and cultured for 28 days, in three biological replicates. At 1 and 28 days, an aliquot of culture was plated and viable cells that grew into colonies were collected, prepared, and barcode-sequenced. We identified plasmids whose relative barcode abundance was enriched at 28 days compared to the starting library in each strain (at a relaxed FDR cutoff of 0.1 given noise in the assay, see Methods). A positive enrichment suggests a fitness benefit to cells that caused them to rise in frequency over time.

We identified 62 genes that reproducibly increased in the pool over 28 days versus the starting library in YPS1009_Chr12 and that were at least 2-fold more enriched than in the euploid analyzed in the same way (see Methods). We reasoned that genes that impact the health of aneuploid cells early in the time course would be already enriched at 1 day of culturing (Fig 2B, orange gene count), whereas genes that specifically impact life span would be more enriched at 28 days (Fig 2B, pink gene count). Of the 62 genes identified, 22 were more enriched (>2-fold) in abundance at 28 days compared to 1 day, implicating a specific impact on life span (S1 Data). Nearly half of these genes encode proteins localized to the mitochondria, whose function is critical for quiescence and life span but known to be defective in aneuploid yeast and mammalian cells [70–76]. The other 40 genes were enriched over the starting library at 28 days but also at 1 day of culturing (see Methods), suggesting an early impact on culture growth and cell health. Among these are genes already linked to life span from other studies, including sirtuin Hst2 tenuously linked to life span [77–79], several genes involved in autophagy (*ATG12*, *POR1*), which is required for healthy aging [80,81], and others discussed more below (*SGT1*, *SCP1*) [82,83].

Among the most intriguing on the list of 40 genes was *RQC1*, a component of the RQC pathway that resolves stalled ribosomes [38,39,84,85]. This was interesting because aneuploid strains are sensitive to translational inhibitor nourseothricin (NTC) that binds the ribosome to disrupt translation [13,25,86–88]. Given that translation defects increase with cellular age [47–49], these results raised the possibility that chromosome duplication induces defects in the RQC pathway.

### The RQC pathway is directly involved in aneuploidy-dependent quiescence defects

We explored the impact of specific perturbations to the RQC pathway. Both Rqc1 and Ltn1 are stoichiometrically limiting in yeast and mammals [38,89,90]. Remarkably, the defect in cell-cycle arrest was significantly alleviated simply by duplicating *RQC1* or *LTN1* on a plasmid, in all aneuploids tested: significantly more cells in each culture arrested as unbudded cells by 2 days (Fig 2C). Conversely, deleting these genes made the arrest phenotypes worse (S2A Fig). We tested other players in the RQC pathway as well. Previous work showed that deletion of *RQC2* can actually alleviate the stress of a dysfunctional RQC pathway. This led to the suggestion that CATylated intermediates may be toxic [45,54,91]. Indeed, deletion of *RQC2* alleviated the arrest defect. Furthermore, expression of the *rqc2 D98Y* mutant unable to add CAT tails [42,43] complemented the defect to levels that are statistically indistinguishable from the full *RQC2* deletion (*p* > 0.1, Fisher’s exact test, Fig 2D). We also tested the upstream sensor of stalled ribosomes, Hel2, which participates in multiple translational quality control pathways [92–94]; duplication of *HEL2* also alleviated the arrest defect to varying degrees. We were unable to test the impact of Hel2 deletion, since the YPS1009_Chr4 *HEL2* deletion strain was not viable and YPS1009_Chr12 *hel2Δ* cultures did not maintain aneuploidy, suggesting that these aneuploids are especially reliant on Hel2. Together, these results show that all of the tested players in the RQC pathway affect cell-cycle arrest in aneuploids entering quiescence. Augmenting RQC genes also affected other aneuploidy quiescence defects, improving morphology defects of YPS1009_Chr14 and _Chr15 cells (S2B Fig) and increasing final optical density and/or cell densification of YPS1009_Chr4 and _Chr12 aneuploids (S2C and S2D Fig).

An important question is if augmenting the RQC pathway improves aneuploid life span. We devised a more sensitive single-cell, live-dead assay to assess viability by day, in aneuploid cells over-expressing *RQC1* or *LTN1* as representatives. Increasing either gene improved viability in all three aneuploids scorable by this method, to comparable levels (Fig 2E, *p* < 1 × 10^−5^, two-way ANOVA comparing strains across timepoints to the isogenic control, S2 Table). Viability in aneuploids with the empty vector began declining as early as day 7 of culturing. In contrast, *RQC1* or *LTN1* over-expression increased viability compared to the empty vector out to 18 days (in the case of Chr4*-RQC1, -LTN1*) or 21 days (for Chr14-*RQC1*, Chr12-*RQC1*, *LTN1*, *p* < 0.05, one-tailed *T* test comparing each time point to the control, see S2 Table). Overproduction of the genes also increased the total cell number of YPS1009_Chr12 cells (Figs 2F and S2C), explaining why *RQC1* produced a fitness benefit at day 1 of the genomic screen. The impact on viability is clearly partial, since none of the plasmids increased viability to euploid levels. Nonetheless, over-producing single RQC subunits improves aneuploid health and viability in all aneuploids tested.

### Aneuploid cells accumulate stalled ribosomes and RQC defects

**Fig 3:**
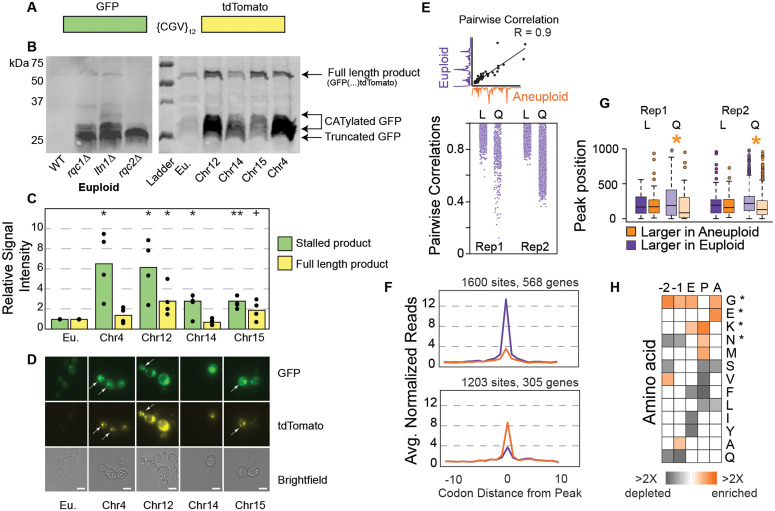
Aneuploids show signs of RQC defects. (**A)** Ribosome stalling reporter, see text. (**B)** Representative anti-GFP Westerns of euploid (left) and aneuploid strains (right) carrying the ribosome stalling reporter, cultured for 4 days and normalized by cell number. (**C)** Average and individual data points (*n* = 4) of relative RQC signal normalized to Ponceau S (see Methods). Asterisk, *p* < 0.05, replicate-paired *T* test compared to euploid. (**D)** Representative GFP (top), tdTomato (middle), or bright field microscopy. Scale bar, 5 µm. (**E)** Ribosome traces (codon-level occupancy normalized to transcript-body occupancy, see Methods) were compared for each mRNA in euploid and aneuploid cells, and the correlation between traces was calculated as shown in the cartoon. The distribution of pairwise correlations comparing euploid and YPS1009_Chr12 cells is shown for cells in log phase (L) or entering quiescent (Q, day 4). (**F)** Average ribosome occupancy (as described in E) for peaks with higher occupancy (FDR < 0.05) in euploids (purple, top) or in YPS1009_Chr12 (orange, bottom). (**G)** Distribution of codon positions for peaks higher in euploids (purple) or in aneuploids (orange). Asterisk, *p* < 0.05, Wilcoxon test. Some outliers are omitted from display. Replicate distributions were not different from one another (*p* > 0.1 in all cases). (**H)** Amino acids (rows) at aneuploidy-increased ribosome occupancy sites that are enriched or depleted compared to the YPS1009 proteome, shown for statistically significant sites (FDR < 0.05, Fisher’s exact test). Asterisk, enrichments seen previously in yeast ribosome stall sites [47]. The data underlying this figure can be found in S2 Table.

Results above point to a problem in RQC during quiescence in aneuploids. We therefore tested if aneuploid cells show signatures of ribosome stalling. We used a reporter with a stall-inducing stretch of 12 arginine codons between GFP and tdTomato coding sequences (Fig 3A). Healthy cells can readily dismantle and degrade ribosomes stalled on the reporter. We confirmed that euploids lacking *RQC1* or *LTN1* accumulate a smeared product consistent with CATylated GFP (Fig 3B). As expected, the absence of Rqc2 in euploids produced a crisp GFP band without the diagnostic smear of CATylation.

In contrast to euploid cells, all of the wild-type aneuploids tested accumulated significantly more RQC intermediates by both Western analysis (Fig 3B and 3C) and microscopy (Figs 3D and S3). Interestingly, the more sensitive microscopy data indicate that these defects accrue over time: GFP signal indicative of stalled translation on the reporter was low in log-phase cells but accumulated as early as 1 day of culturing in all four aneuploids tested, preceding the budding defects seen at day 2 (S3 Fig). Interestingly, many cells accumulated both GFP and tdTomato signal by day 4 of culturing, reflecting translation through the stall site to produce full-length protein (Figs 3D and S3). These results suggest that aneuploid cells may suffer from different RQC defects at different chronological ages (see Discussion).

To confirm the stalling reporter results, we performed replicate ribosome footprinting on euploid and the YPS1009_Chr12 aneuploid as a representative, in log phase and after 4 days of culturing. Ribosome occupancy at each codon was calculated as codon-level read count normalized to gene-body read count as done previously [47]. In exponentially growing cells, the transcript-specific traces of ribosome occupancy were generally very similar between euploid and aneuploid cells (Fig 3E). In contrast, many traces of ribosome occupancy were reproducibly poorly correlated in euploid and aneuploids entering quiescence, indicating differences in ribosome pausing on the same transcripts. We identified codons with statistically significant differences in normalized ribosome occupancy (FDR < 0.05, Fisher’s exact test, see Methods). We found 1,600 sites in 568 transcripts that displayed higher ribosome occupancy in quiescent euploid samples, whereas 1,203 sites in 305 transcripts with higher occupancy in the quiescent aneuploid (Fig 3F). While there was a slight enrichment for Chr12 transcripts over those from other chromosomes (*p* = 0.03, hypergeometric test compared to measured RNAs), these comprised only 10% of affected RNAs. Many transcripts showed multiple peaks with opposing effects, such that one site on the transcript had higher occupancy in the aneuploid but other peaks on the same transcript were larger in the euploid. While investigating this, we noticed that aneuploid-enhanced peaks tended to occur nearer to the start of the transcript (Fig 3G, *p* < 0.05, Wilcoxon rank-sum test, S4 Fig), for reasons that will require further study to dissect. Sites with higher ribosome occupancy in the quiescent aneuploid were enriched for residues previously associated with ribosome stalling in yeast, including lysine and asparagine at the P site and glycine and glutamate at the A site (Fig 3H) [47]. Importantly, peaks that were called significantly different between log-phase strains were typically subtle in magnitude (S4B Fig) and showed no significant difference in associated sequences (FDR > 0.05 at all sites). Thus, aneuploid YPS1009_Chr12 cells show increased ribosome occupancy at sequences known to induce ribosome stalling, but only during quiescence, consistent with results using the ribosome stalling reporter.

### Inducing ribosome stalling in euploids disrupts quiescence and life span

**Fig 4:**
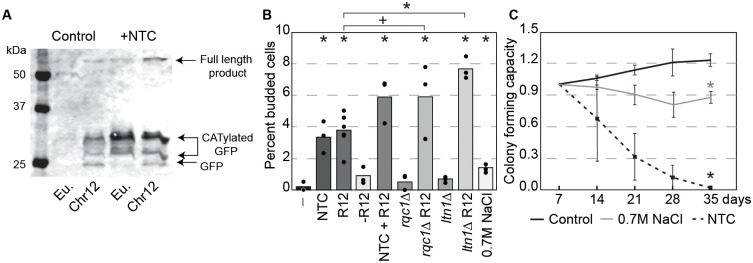
Induction of ribosome stalling disrupts quiescence and life span in euploid cells. **(A)** Representative anti-GFP western blot of euploid (Eu) and YPS1009_Chr12 cells with the stalling reporter, as described in Fig 3B, cultured for 4 days with and without low-dose (1 µg/mL) NTC. **(B)** Average and individual data points of percent budded cells in wild-type, *rcq1∆,* or *ltn1∆* euploid cells exposed to 1µg/mL NTC, carrying the ribosome stalling reporter (“R12”) or non-stalling control reporter (“-R12”), or exposed to 0.7M sodium chloride as a control. Asterisk, *p* < 0.05, +, *p* < 0.1, compared to WT or as indicated, *n* = 3–6; Fisher’s exact test applied to cell count data.(**C)** Average (*n* = 3) and standard deviation of colony forming capacity in euploid cells normalized to 7 days. Asterisk, *p* < 0.05 compared to untreated control at 35 days; *T* test. The data underlying this figure can be found in S2 Table.

If problems managing ribosome stalling drive quiescence defects in aneuploids, then increasing the level of ribosome stalling in euploids should cause similar defects. Indeed, this was the case. Low doses of NTC induce ribosome stalling, evident by increased RQC intermediates from the stalling reporter in normalized cell counts (Fig 4A). This dose of NTC was enough to significantly increase the number of euploid cells that remained budding at day 3 (Fig 4B). Remarkably, simply over-expressing the RQC reporter had the same effect, whereas expressing a control protein without the stall-inducing sequence (“-R12”) did not. Expressing the reporter in conjunction with NTC treatment or in the absence of *RQC1* or *LTN1* significantly exacerbated the defects, whereas stressing cells with sodium chloride as a control had only a minor effect (Fig 4B). Together, these results indicate that increasing ribosome stalling in euploid cells induces the arrest defect. It also slightly but significantly disrupted cell densification compared to the euploid control (S5 Fig). In contrast, deleting *RQC1* or *LTN1* from the euploid had only a minor effect on budding, unless cells also carried the RQC reporter. Thus, exacerbated stalling is required to produce the arrest defect. Consistent with the model that increased stalling shortens life span, these treatments in euploid cells significantly shortened life span to much greater levels than a salt-stress control (Fig 4C). Together, these results show that it is the increase in or perhaps quality of ribosome stalling that affects quiescence and life span, rather than a defect managing basal levels of stalling seen in the euploid.

Interestingly, we noticed that NTC treatment and/or the stalling reporter significantly exacerbated defects seen in aneuploid cells, including odd morphologies of starved cells entering quiescence. In fact, NTC treatment of YPS1009_Chr12 induced a small number of bi-lobed cells characteristic of YPS1009_Chr14 entering quiescence, and in a few instances produced cells in which nuclear division occurred perpendicular to the division plane (S6A Fig). These morphologies are reminiscent of those caused by defective Cdc34, the E2 ubiquitin conjugase of the SCF complex that marks cell-cycle regulators for timed degradation by the ubiquitin-proteasome system [95–97].

### Genes linked to ubiquitin metabolism alter aneuploid arrest phenotypes

**Fig 5:**
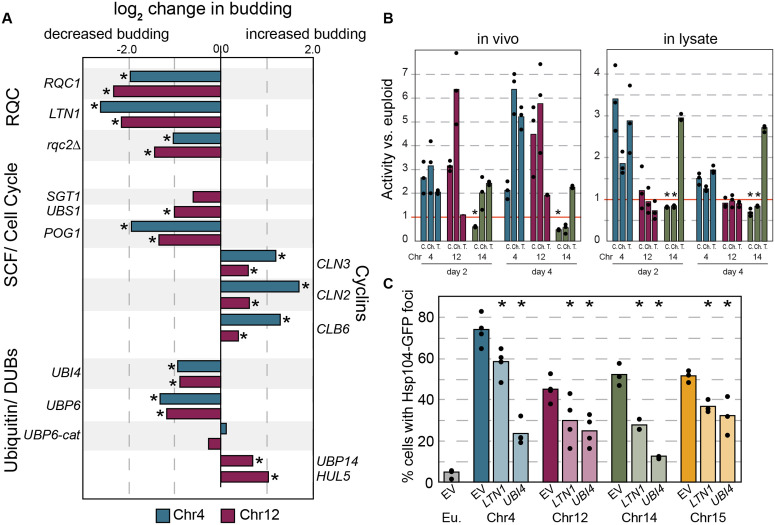
Genetic perturbation influences aneuploidy phenotypes. **(A)** log_2_ change in % budded cells for Chr4 (blue) or Chr12 (magenta) aneuploids with denoted genes vs. empty vector (data for RQC subunits reprinted from Fig 2 for comparison). Other tested genes had no significant effect (*AFG3, ATG12, GRR1, OTU1, PCL2, PRE1, RPN11, YGP1, LEE1)*. Not all genes were tested in YPS1009_Chr4. (**B)** Average and individual data points (*n* = 3) of proteasome activity measured in permeabilized cells (left) or lysate (right) in aneuploids relative to the paired euploid, for caspase (C), chymotrypsin (Ch), or trypsin (T), see Methods. Asterisk, *p* < 0.05 for samples with less activity than euploid. (**C)** Percent cells with Hsp104-GFP foci (*n* = 3–4) for aneuploids harboring indicated gene duplications. Asterisk, *p* < 0.05 compared to empty vector control, Fisher’s exact test applied to count data. The data underlying this figure can be found in S2 Table.

To further dissect how RQC defects could impact cell cycle arrest, we returned to hits from our screen in YPS1009_Chr12. Several hits were linked to SCF-dependent protein degradation, including Cdc34 regulator *UBS1* [98,99], chaperone *SGT1* that associates with SCF [100], and *POG1* that has been implicated in G1/S regulation and can suppress defects in E3 ubiquitin ligase Rsp5 [101–103]. Duplication of *UBS1* and *POG1* partly alleviated the arrest defect in YPS1009_Chr4 and/or YPS1009_Chr12, whereas *SGT1* had a mild effect but missed the significance cutoff (Fig 5A). This raised the possibility that a defect in SCF and/or protein degradation may disrupt turnover of important cell-cycle regulators. Nearly all budded cells lacked nuclear Whi5-GFP consistent with elevated Cln3 activity (S6B and S6C Fig). If cyclin degradation is disrupted in this strain, then over-expression of cyclins may exacerbate phenotypes. Indeed, duplicating *CLN3* or downstream G1 and S phase cyclins *CLN2* or *CLB6* statistically significantly increased budding in both YPS1009_Chr4 and YPS1009_Chr12 aneuploids, with only a weak effect on euploid cells (Figs 5A and S6D). These results raise the possibility that cyclins including Cln3 are not properly degraded in aged aneuploids and show that increased cyclin gene copy exacerbates defects. Cyclins are notoriously challenging to detect by western blot; however, we were able to detect degradation products of HA-tagged Cln3, but only in quiescent YPS1009_Chr12 aneuploid and not quiescent euploid cells (S6E Fig). Validation of the model that Cln3 cyclins are not fully degraded will require further study.

One possibility is that chromosome amplification disrupts inherent proteasome function, as previously proposed in the aneuploidy-sensitized laboratory strain [104,105]. However, this does not appear to be the case in the wild strain background: we measured three separate proteasome activities in live cells and cell lysates using luminescent reporters that do not require ubiquitination for degradation [106]. Overall, aneuploids tested did not show grossly lower proteasome activities compared to euploids, and several activities were substantially higher than euploid (Fig 5B). Aside of YPS1009_Chr14, which showed slightly lower levels of chymotrypsin and caspase activity, the other scorable aneuploids showed activity equal to or even greater than wild-type cells, with some variation depending on the assay. (Chr15 was not tested due to challenges normalizing cell numbers). Furthermore, none of the aneuploids was more sensitive than the euploid to proteasome inhibitor MG132 in this strain background, in log phase or based on final cell number in saturated cultures (S7A and S7B Fig). Thus, inherent defects in proteasome function or assembly are unlikely to explain the common quiescence defects seen across multiple aneuploid strains.

However, in testing possible models, we found that duplication of the stress-induced polyubiquitin gene *UBI4* significantly alleviated the aneuploid arrest defect in Chr4 and Chr12 aneuploids, as did duplication of deubiquitinase (DUB) *UBP6* that is important for ubiquitin recycling (Fig 5A). The alleviating effect of *UBP6* duplication is the opposite effect reported for the sensitized W303 strain, where deletion of *UBP6* provided a benefit, reportedly by relieving proteasome inhibition that occurs through a separable Ubp6 domain [104,105,107]. To distinguish between these functions, we tested catalytically inactive ubp6-C118A that can still inhibit proteasomal processivity but lacks ubiquitin recycling activity. This mutant did not mitigate aneuploid arrest defects, indicating that the DUB activity is required (Fig 5A). In contrast, duplicating ubiquitin ligase *HUL5*, which antagonizes ubiquitin recycling by extending ubiquitin chains and increasing proteasome processivity, exacerbated arrest defects (Fig 5A). Duplication of a different DUB, *UBP14,* which disassembles unanchored ubiquitin chains and on specific targets [108], increased defects. Together, these results suggest an issue with ubiquitin recycling, availability, and/or metabolism, rather than an inherent proteasome defect, that underlies aneuploid phenotypes. Notably, none of the gene duplications tested alleviated the arrest defect to the same level as *RQC1* or *LTN1*.

### Proteostasis stress is alleviated by duplication of RQC or ubiquitin

Trisomy 21 in humans and chromosome amplification in sensitized W303 yeast is associated with increased protein aggregation, although the source of proteostasis stress is not known. We previously showed that wild aneuploid yeast strains do not show signs of proteostasis dysfunction unless stressed by *SSD1* deletion or treated with translational inhibitor NTC [13,25]. Here we found that aging also induces protein aggregation in wild aneuploids: at 7 days of culturing, 50%–70% of aneuploids with extra Chr4, Chr12, Chr14, or Chr15 harbored foci of protein disaggregase Hsp104 (Figs 5C and S8), compared to ~5% of euploid cells. The proportion of aneuploids with such aggregates decreased substantially upon duplication of RQC gene *LTN1* or polyubiquitin *UBI4* as representative RQC and ubiquitin-affecting genes (Fig 5C). We confirmed in YPS1009_Chr4 and _Chr12 aneuploids that deletion of *RQC2* also significantly reduced Hsp104-GFP signal (S7D Fig). These results suggest that age-induced protein aggregates can be alleviated by duplication of limiting RQC subunit Ltn1 or polyubiquitin, or deleting *RQC2*.

**Fig 6:**
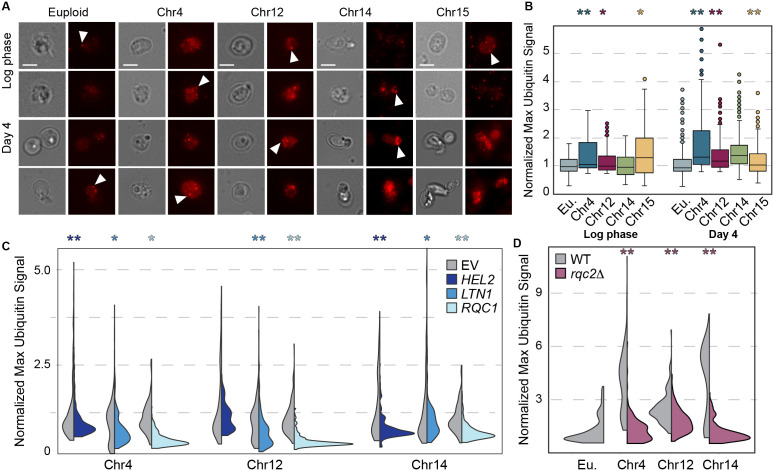
Aneuploids exhibit altered ubiquitin distributions. (**A)** Representative brightfield and fluorescent images of euploid and aneuploid cells at log-phase and day 4 stained with fluorescent anti-ubiquitin antibody. Scale bar, 5 µm. (**B)** Distributions of maximum pixel intensity of ubiquitin signal per cell in euploid and aneuploid cells at log-phase and day 4, normalized to median signal intensity of paired euploid control. * *p* < 0.05, ** *p* < 1 × 10^−10^ Wilcoxon rank-sum test. (**C)** As in B but using split violin plots to show distributions of aneuploids harboring *HEL2, LTN1,* or *RQC1* overexpression plasmid vs. paired empty vector (EV) controls done side-by-side. (**D)** As in C but for wild type (gray) or *rqc2Δ* cells, according to the color key (D). * *p* < 1 × 10^−3^, ** *p* < 1 × 10^−10^ Wilcoxon rank-sum test. Some outliers (for Chr4 in B and Chr14 *LTN1* EV in C) are omitted from display, see S2 Table for counts. The data underlying this figure can be found in S2 Table.

We considered several models for how RQC defects could perturb ubiquitin homeostasis. One possibility is that protein aggregation triggered by RQC defects depletes free ubiquitin in aged aneuploids, as proposed in several neurological disorders associated with aggregates [109–111], especially since non-mitotic cells rely on ubiquitin recycling for proteostasis maintenance [112]. But western blots of bulk-culture lysates revealed that monoubiquitin is still visible in aged aneuploids (S7C Fig), indicating that at least some cells in the culture harbor free ubiquitin (see Discussion). We next asked if ubiquitin localization was different in aneuploid cells using fluorescence microscopy. Indeed, the ubiquitin profile was markedly different in aged aneuploids (Fig 6A). Whereas ubiquitin was evenly distributed across most of the aged euploid cells, the majority of aged aneuploid cells showed significantly brighter ubiquitin signal, often in discrete puncta (Fig 6B). Importantly, duplication of *RQC1, LTN1 and HEL2, or deletion of RQC2,* significantly reduced ubiquitin signal in all four aneuploids tested (Fig 6C and 6D). Thus, the impact of RQC augmentation on the ubiquitin localization phenotype parallels its impact on cell-cycle arrest, cell densification, Hsp104 aggregates, and viability in aging aneuploid cells. We were unfortunately unable to study co-localization of ubiquitin and RQC stalling products due to challenges with multi-antibody microscopy in fixed quiescent cells, thus leaving several possible causal models discussed below.

## Discussion

Shortened life span is a hallmark of DS and has also been observed in aneuploidy-sensitized laboratory yeast and yeast with higher ploidy [2,66,113]. Here, we show that chromosome amplification produces multiple aging signatures, including defects in quiescence, accumulation of proteostasis stress, and shortened life span, across affected chromosomes but influenced by chromosome-specific effects. Signatures of premature aging have also been observed in other aneuploid syndromes, including early senescence and protein aggregation in human trisomy 13 and 18 [114,115]. Together, this strongly suggests that premature aging is a generalizable consequence of chromosome amplification conserved across species and affected chromosomes. The mechanism underlying premature aging in DS has been a mystery. Our results suggest that defects in RQC may contribute: aneuploid yeast strains studied here accumulate RQC intermediates, show aberrant ribosome profiles, and harbor aneuploidy-associated protein aggregates. Several of these aneuploidy phenotypes, including defective cell-cycle arrest and altered ubiquitin distributions among others, can be partly corrected simply by augmenting the RQC pathway. Conversely, inducing ribosome stalling in euploid yeast accelerates aging and decreases life span, confirming a causal link. Healthy aging is already associated with a decline in both translational fidelity and proteostasis management across organisms [48,116,117]. Our work here adds to a growing body of evidence that chromosome amplification accelerates that decline.

Why does chromosome amplification cause RQC defects? We propose that simply having too many translating mRNAs in the cell is enough to overwhelm the RQC pathway, and that early defects in RQC catalyze a series of later problems. We and others showed that, although RNAs from some amplified genes are dosage compensated in yeast, most are not—thus aneuploid cells have proportionately more mRNAs for hundreds of amplified genes [7,13–15,118]. Ribosome profiling shows a median of 1.7× higher read counts mapping to transcripts from the duplicated chromosome in both log and quiescence phase, as expected if the mRNAs are translated at equivalent levels per molecule as in the euploid. Furthermore, all aneuploids tested here have a defect silencing the transcriptome on quiescence entry. Thus, aging aneuploid yeast harbor substantially more RNAs than the corresponding euploid. Many of these mRNAs are likely translated, since we find they remain associated with ribosomes in YPS1009_Chr12. Given that Ltn1 and Rqc1 are sub-stoichiometric to the ribosome [38,89,90], an over-abundance of translating mRNAs could titrate RQC subunits, leading to the accumulation of RQC intermediates early during the aging process. Interestingly, the nature of RQC defects appears to change over time: although early (day 1–2) signs suggest a defect clearing stalled ribosomes, later (day 4) signatures suggest a wholesale failure to initiate the pathway, since cells accumulate full-length reporter protein. One possibility is that an early Ltn1/Rqc1 defect goes on to create other RQC failures over time. Detailed biochemistry will be required to elucidate molecular details, but of interest in this paper is the physiological impact: over-expressing RQC subunits in aneuploids alleviates multiple aging phenotypes, while inducing ribosome stalling in the euploid creates them.

**Fig 7:**
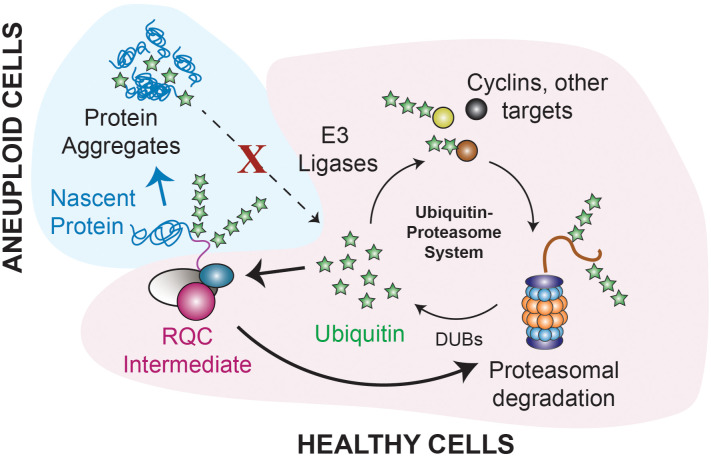
Model for RQC impacts on aging in aneuploid cells. Healthy cells maintain ubiquitin stasis as part of the Ubiquitin-Proteasome System (UPS). We propose that defects in RQC either produce or indirectly cause protein aggregates that sequester ubiquitin, thereby depleting the pool of accessible ubiquitin, see text for details.

A second question is how RQC defects cause cellular decline. We propose that RQC intermediates lead to defects in proteostasis management that accelerate aging (Fig 7). Aneuploids entering quiescence show an increase in Hsp104 foci indicating protein aggregates and abnormal distribution of ubiquitin, and both phenotypes are partly corrected by augmenting the RQC pathway. One possibility is that aggregates either comprise or are indirectly caused by CATylated RQC intermediates reportedly prone to aggregation [43–46] (Fig 7). Indeed, deleting *RQC2* or expressing the CATylation-defective rqc2 D98Y mutant [43] alleviated tested quiescence defects. Why the aggregates are not turned over is less clear. We found no evidence for an inherent proteasome defect (Figs 5B and S7A, S7B), although it is possible that proteasomes are overwhelmed in ways we cannot detect. Another possibility is a deficit in autophagy, which degrades aggregates but is disrupted in some aneuploid lines and cancers [11,119,120]. Both processes depend on ubiquitin, which our results show is at play. Overexpressing polyubiquitin decreased Hsp104 foci and alleviated the arrest defect. Furthermore, duplicating DUB Ubp6 that recycles ubiquitin improved arrest, whereas expressing Hul5 that counteracts Ubp6 by extending ubiquitinated chains exacerbated the defect (Fig 5A). A simple hypothesis is that aging aneuploids have a defect in ubiquitin availability or distribution that perturbs ubiquitination of specific impactful proteins. Unlike dividing cells that synthesize ubiquitin during growth, post-mitotic quiescent cells rely on ubiquitin recycling to maintain pools [112,121]. Protein aggregates in post-mitotic neurons are known to sequester ubiquitin, which is proposed to decrease the pool of free ubiquitin and thus limit both proteasomal and autophagosomal degradation [109–111]; multiple neurodegenerative diseases are also associated with RQC defects [109,122–124]. Lysate from bulk aneuploid yeast appeared to express mono-ubiquitin signal (S7C Fig); however, it is possible that free ubiquitin is either locally or globally depleted in the subset of cells with substantial RQC defects. This could disrupt which proteins are targeted for degradation, either globally or at specific targets with large downstream effects. We found the ubiquitin distribution substantially altered in all aneuploid yeast strains tested (Fig 6), which in all cases was alleviated by augmenting the RQC pathway. This strongly suggests that RQC failure is an upstream event that impacts ubiquitin stasis, which produces broader downstream phenotypes. Interestingly, several DUBs and E3 ligases have been implicated in aneuploidy already, in sensitized W303 yeast [26,66,104,105] and DS [35,125,126], consistent with an underlying difference in ubiquitin stasis. It is worth noting that ubiquitin is also important for translational fidelity, since yeast with defective ubiquitin metabolism are sensitive to translational inhibitors like NTC [127–129]. Thus, inaccessibility of ubiquitin could further exacerbate translational defects in aneuploid cells.

While we characterized multiple aneuploid defects during the aging process, most were heterogeneous in that they affected only a subset of cells. Yet, shortened life span was universal to all cells in the culture. We propose that aneuploid cells may operate around a threshold of biosynthesis errors, below which they can function but over which they experience cellular collapse [13]. Stochastic fluctuations push many individual cells past the point of no return to a healthy state. This model could explain the previously observed heterogeneity in aneuploid phenotypes [130,131], but could also produce multiple trajectories of premature aging [132,133]. Importantly, augmenting RQC improved aneuploid viability and extended life span but only partially. It is likely that other factors, including mitochondrial function that is important for yeast life span and defective in both yeast and DS aneuploids are involved [13,76].

Our results point to a generalizable effect of chromosome amplification on translational fidelity, common across multiple affected chromosomes studied here. But the genes on the affected chromosomes clearly matter, contributing chromosome-specific effects like aberrant cellular morphology, densification defects, and reduced post-diauxic growth (Figs 1 and S1). The same type of interplay may be true in people with DS. For example, human Chr21 carries APP that underlies amyloid plaques in Alzheimer’s disease; yet a 50% increase in APP abundance may not fully explain early-onset disease in people with DS [134,135]. Amplified gene DYRK1A is also proposed to contribute to DS phenotypes, including those linked to aging in some tissues [136,137]. A generalizable contribution of chromosome amplification could exacerbate single-gene effects, including genes with tissue-specific roles. An important avenue of study is to test the extent that generalizable defects in RQC and ubiquitin stasis contribute in aneuploid human cells.

## Methods

### Strains and plasmids

Strains used in this study are listed in S1 Table. YPS1009 aneuploids were generated in Rojas and colleagues [57]. Most chromosome duplications are stable for many generations; maintenance of aneuploidy was confirmed periodically by plating cultures from each experiment onto synthetic complete media with selection for marked chromosomes (SC −HIS +NTC). Gene deletions were generated by homologous recombination of the Hph-MX drug resistance cassette into the designated locus, followed by diagnostic PCR to confirm correct integration and absence of the target gene. Aneuploidy was confirmed and periodically checked through diagnostic qPCR of one or two genes on the affected chromosomes normalized to a single-copy gene elsewhere in the genome – normalized ratios close to 2 reflect gene duplication, and ratios between 1.2 and 1.8× indicated partial loss of aneuploidy in the cell population. *HSP104*-GFP was generated by integrating a GFP-ADH2 terminator::HIS3 cassette into the *HSP104* locus [138] via homologous recombination. Aneuploid Hsp104-GFP strains were then generated through mating and dissection, crossing AGY1970 to relevant aneuploids. The GFP-{CGV}12-tdTomato stalling reporter was generated by PCR sewing and cloning the generated fragment into a KAN-marked CEN plasmid (pJH2). Unless otherwise noted, plasmids used in this work were from the MoBy 1.0 plasmid library [69]. The *RQC2* plasmid was constructed by PCR amplification of the coding sequence and flanking regions (450 bp upstream and 200 bp downstream) of *S. cerevisiae RQC2*, then cloned into the MoBy 1.0 vector (KanMX marked). The *RQC2_D98Y* mutation was constructed by site-directed mutagenesis using Q5 Site-Directed Mutagenesis Kit (NEB).

### Growth conditions

Unless otherwise noted, all experiments were performed in rich YPD (Yeast extract, Peptone, Dextrose) medium. Quiescent cultures were generated by inoculating liquid YPD medium at an optical density (OD_600_) of 0.05. Cultures were allowed to reach saturation and then maintained at 30 °C in a shaking incubator for the number of days indicated, with no nutrient supplementation. Maintenance of aneuploidy was verified by plating an aliquot of aneuploid cultures onto rich YPD plates, then replica plating to SC −HIS +NTC after 24 hours to determine maintenance of the two chromosome markers. Aneuploidy was also periodically verified through diagnostic qPCR as described above. Where indicated, cells without the NAT-MX resistance cassette were treated with 1 ug/mL NTC after cells reached mid-log phase.

### Microscopy

#### Bud indexing.

Cultures were grown in YPD for 2 days and fixed with formaldehyde as described previously [139]. Cells were stained with DAPI using NucBlue ReadyProbes (ThermoFisher, R37606) for 20 min at room temperature to stain DNA. Images were acquired as z-stacks every 0.2 mm using an EVOS FL Auto 2 with a 100× Nikon oil immersion objective equipped with an EVOS DAPI light cube. Cells were scored as budded or unbudded based on morphology and DAPI signal, using a conservative approach that may underrepresent budded cells. Cells were scored budding if they showed budded morphology, lacked septum in the bright-field image, and harbored a bud that either lacked DAPI signal (S-phase to early G2 buds) or possessed bar nuclei (late G2 to M-phase buds). Late-stage buds were likely undercounted by this method, to avoid counting unbudded G1 cells clumped together. A minimum of 5 diverse xy positions and 100 cells were scored per replicate. A minimum of 3 biological replicates were conducted, and statistical significance was assessed by Fisher’s exact test on count data pooled across three replicates, all of which showed consistent trends. Percent budded cells shown in the figures was calculated as a percent cells scored as budded ((# of budded cells/# of cells scored) * 100). Indexing was conducted in the same way for indicated strains carrying plasmids, except cells were grown in YPD with G418 to select the plasmids. Morphology differences shown in Fig 1 were scored manually by counting (i) for YPS1009_Chr14, “peanut” shaped cells with a round nucleus centered between the lobes, or (ii) for YPS1009_Chr15 scoring all cells with multiple lobes, both shown in Fig 1C. Counts for all microscopy data are available in S2 Table.

#### Live cell microscopy.

After culturing for 4 days, live cells were deposited onto plain glass slides. Images were acquired as z-stacks every 0.2 mm using an EVOS FL Auto 2 with a 100× Nikon oil immersion objective. GFP and tdTomato images were acquired with EVOS GFP and RFP light cubes, respectively. Fluorescent images represent collapsed Z-stacks, and brightfield images represent one z-plane.

#### Immunofluorescence.

Cells were harvested during exponential phase (OD_600_ 0.4–0.6) or 4 days after start of culturing and fixed with 4% formaldehyde for 15 min at 30C followed by centrifugation. Cells were spheroplasted with zymolyase then treated with 0.1% SDS in buffer A (100 mM Tris, pH 8 1M sorbitol) for 10 min. After washing with buffer A, cells were then plated onto a 96-well black-walled plate with a poly-L-lysine-coated coverglass bottom (Cellvis). After 30 min of incubation with blocking buffer (50 mM Tris pH 8, 150 mM NaCl, 1% nonfat dry milk, 0.5 mg/ml BSA, 0.1% Tween 20), cells were exposed to anti-ubiquitin antibody (Milipore Sigma, MAB1510) in blocking buffer overnight at 4 °C. After washing with blocking buffer, cells were exposed to anti-mouse Alexa Fluor 647 antibody (Life Technologies, A21235) for 1 hour at room temperature. After washing with blocking buffer, ProLong Gold Antifade Mountant (ThermoFisher, P36934) was applied to each well. Images were acquired as Z-stacks every 0.2 mm using an EVOS FL Auto 2 with a 100× Nikon oil immersion objective. FIJI (imageJ) was used to determine ubiquitin signal intensity. Brightfield images were used to generate cell masks and maximum pixel intensity of ubiquitin immunofluorescence was computed for each cell, to capture foci. Trends were very similar studying mean signal intensity per cell.

#### Viability assay.

An aliquot of culture was harvested on each indicated day, stained with 0.5 µM propidium iodide (Sigma) for 20 min, plated onto a 96-well black-walled plate with a poly-L-lysine-coated coverglass bottom (Cellvis), then imaged with an Evos FL Auto 2. Brightfield and fluorescent images were acquired with EVOS RFP light cube. Viability is calculated as the # of PI-negative cells/ # of cells present in brightfield image.

### Cell-density fractionation

Density gradient sedimentation and fractionation of stationary phase cultures was performed using Percoll (Sigma, P1644) as described previously [140]. Gradients were split evenly between two fractions. Fractions were collected using 18-gauge needle and 10 ml syringe, harvesting the heavy fraction first, then washed once in PBS, and resuspended in 1 mL of PBS. Fractions were quantified using a hemacytometer and optical density.

### Chronological life span assay

Chronological life span in Fig 1G was determined by plating for colony-forming capacity over time. At various times over long-term growth, an aliquot of culture was harvested, OD_600_ measured, and cells diluted serially to a 40,000× dilution, which was spread on YPD plates. Plates were incubated for 48 hours, and viable colonies were counted using ImageJ Colony Counter Plug-in (ImageJ) to quantify colony-forming units. Colony-forming capacity was calculated as colony-forming units divided by optical density measured at the indicated day.

### RNA sequencing

RNA-seq was performed using total RNA isolated from log-phase and quiescent cultures. Cultures were started at OD_600_ 0.05. Log-phase cultures were harvested after precisely three generations. Day 1, 3 and 7 cultures were harvested 24, 72, and 168 hours after log-phase cultures were harvested. OD-normalized samples were pelleted by centrifugation and flash frozen with liquid nitrogen and maintained at −80 °C until RNA extraction. Samples were mixed with a defined number of flash-frozen *Sz. pombe* cells before RNA extraction, to later serve as per-cell normalization. Total RNA was extracted by hot phenol lysis [141]. Mechanical disruption was required to efficiently lyse quiescent cells: 425–600 µM glass beads (Sigma, G8772) were added to samples in phenol-lysis buffer such that glass beads accounted for 1/3 of total sample volume. Greater than 50% of empty space was maintained in sample tubes to ensure efficient lysis. Samples were then vortexed for 1 min in 10 min intervals for 1 hour. rRNA depletion was performed using the Ribo-Zero (Yeast) rRNA Removal Kit (Illumina, San Diego, CA). Libraries were prepared with TruSeq Stranded Total RNA kit and purified using a Axygen AxyPrep MAG PCR Clean-Up Kit (Axygen). Illumina reads were mapped to the S288c genome substituted with SNPs from YPS1009 as called in Sardi and colleagues [142], using bwa-meme [143]. Read counts for each gene were calculated by HT-Seq [144]. Normalization was conducted by setting the slope of *Sz. pombe* reads across samples to 1.0. Statistical analysis of log_2_(fold change) transcript abundances was done in edgeR [145] taking genes with a FDR < 0.05 as statistically significant. Genes shown in Fig 1E were defined as those significantly repressed (FDR < 0.05) in the euploid strain and statistically significantly higher (FDR < 0.05) in all four aneuploids, in at least one time point comparing that aneuploid to the euploid. Hierarchical clustering was performed using Cluster 3.0 [146] and visualized in Java Treeview [147]. Data represent the average of biological duplicate and are available in GEO Accession #GSE269236. Data for Fig 1D are available in S2 Data.

### Genetic screen

Dual-marked YPS1009_euploid (AGY1611) and _Chr12 strains (AGY1612) were transformed with Moby 1.0 low-copy expression library [69] and viable colonies were scraped and frozen at −80 °C. Pooled cells were used to inoculate rich medium and then grown in biological triplicate for 28 days in YPD medium + G418 to maintain plasmids. A portion of each culture was harvested at 24 hours and 28 days. The harvested portions were plated on multiple plates of YPD + G418 to select for cells that were viable and able to form colonies, thus representing quiescent cells that re-entered the cell cycle. After 48 hours of growth, lawns were scraped, collected, and flash frozen. Plasmid DNA was collected from the starting pools, day 1, and day 28 samples using Zymoprep Yeast Plasmid kit (Zymo Research, D2004), with the following changes: 425–600 µM glass beads were with the lysis reagent, and samples were vortexed for 10″ three times during lysis. Samples were incubated on ice for 30 min after adding neutralization buffer. Barcodes were sequenced as previously described [148,149]. EdgeR was used to TMM normalize samples as previously described [145]. Genes with a significant positive log_2_(fold change) in barcode abundance (FDR < 0.05 or FDR < 0.1) at day 28 versus starting pool were considered enriched, *i.e.*, beneficial. We then selected genes with a 2-fold or greater linear difference in enrichment between average YPS1009_Chr12 sample versus average euploid sample, which resulted in 43 candidate genes at an FDR < 0.05 and 62 genes at FDR < 0.1. We focused on the relaxed stringency list to select candidates for downstream validation. Twenty-two of those genes were >2× more enriched at d28 versus d1 in the YPS1009_Chr12 strain, whereas the remaining genes were within 2-fold enrichment at both d1 and d28. Hierarchical clustering was performed on the log_2_(fold change) abundance differences using Cluster 3.0 [146] and visualized using Java TreeView [147]. Data are available in GEO Accession #GSE269237.

### Western blotting

Yeast strains were grown as described above, with the following additions: G418 was used to maintain the ribosome stalling reporter, and cells exposed to NTC were treated with 1 µg/mL NTC after cells reached mid-log phase. 2 OD units were harvested and flash frozen. Samples were lysed in 2× Laemmli buffer with glass beads (500 µM, Sigma). Proteins in Laemmli buffer were resolved in 4%–12% SDS-PAGE gels and transferred to nitrocellulose membrane (0.2 µm, LICOR), unless otherwise indicated. Ubiquitin blotting was performed using 12%–14% Bis-Tris PAGE gels and transferred onto PVDF membrane (0.2 µm, Amersham Hybond LFP). Western blots were developed using anti-GFP (Abcam, ab290) for samples containing the ribosome stalling reporter, anti-ubiquitin (LifeSensors, VU101), or anti-HA (Cell Signaling Technology, C29F4). Blots were developed on a Li-COR Odyssey instrument (Model 9120). Li-COR Odyssey software was used to quantify signal intensity of GFP and ubiquitin antibodies. Repeated attempts to blot against common loading controls were unsuccessful in quiescent cultures; therefore, Ponceau S signal was used to normalize protein loading levels, as performed by others [150]. Ponceau S signal was quantified using FIJI (ImageJ).

### Proteasomal activity

Proteasome-glo Cell-Based Assays (Promega) was used to measure proteasomal activities in vivo and in vitro in lysates. Reagents were prepared according to manufacturer’s instructions. An equivalent number of yeast cells were flash frozen then lysed via vortexing with 500 µM glass beads on ice. Cell lysate and Proteasome-glo reagents were combined 1:1 in an opaque, white-walled 384-well plate (Corning). Luminescence was measured using a Tecan M1000 Pro.

### Ribosome profiling and analysis

Cells were harvested using vacuum filtration with Whatman Nylon filters (Cytiva, 7410-004). Cells were immediately scraped from filters, transferred to eppendorf tubes, and immediately flash frozen. Collection time was <60 seconds. Ribosome profiling was performed by Ezra Biosciences as previously described [151]. Samples were sequenced on an Illumina Novaseq instrument and processed as described in Schuller and colleagues [152] as follows: reads were trimmed with CutAdapt (version 3.5) [153] with command j 8 -g ^GGG -a A{10} -n 2 -m 15 --max-n=0.1 --discard-casava. Reads with poor quality at the 5′ end base (quality score ≤ 10) were removed, reads were mapped to noncoding RNAs from Schuller and colleagues [152], and remaining unmapped reads were mapped to the YPS1009 genome [57] using bowtie2 (version 2.5.1) [154]. The 5′ position of each read was scored, and the P site taken to be at 12 nt into the read [151]. Reads matched well to the expected frame in all samples (see S4 Fig for examples). For each gene, read starts were summed for each position from −72 of the gene ATG and + 60 of each stop codon in the YPS1009 genome. Genes without an annotated ATG were omitted from analysis, as were genes with introns. Read counts were summed for each codon, incremented by 1 pseudocount, and then normalized to the mean read counts per codon (with appropriate pseudocounts) in each gene body, from 60 nt (20 codons) into the gene to 60 nt (20 codons) from the 3′ end as done previously [47]. Genes with at least 50 reads per gene body were retained for further analysis.

The correlation between transcript profiles shown in Fig 3E was taken as the uncentered Pearson correlation for each transcript as measured in euploid and aneuploid, paired by replicate (Fig 3E). Significant differences in ribosome peaks across replicate-paired aneuploid-euploid samples was calculated using Fisher’s Exact test with Benjamini-Hochberg multiple test correction [155], by comparing read count in each sample at a given codon to gene-body read counts for that transcript (# reads at that codon, # reads in the gene body, for euploid versus aneuploid in each replicate separately). Peaks more abundant in the aneuploid were taken as those with FDR < 0.05 and for which the normalized ratio of read counts at that codon was greater in aneuploids; vice versa for peaks more abundant in the euploid. Motif analysis in Fig 3H was performed as follows: codons whose normalized read count differed between euploid and aneuploid samples (FDR < 0.05) were combined across replicates, and peaks more abundant in aneuploids versus euploids were selected. Ten amino acids flanking each peak site were retrieved from the YPS1009 proteasome. The frequency of each amino acid (and stop codon) at each position in the matrix was calculated as the number of occurrences of that amino acid divided by the number of peaks scored. Count and total values were compared at each position in the matrix compared to amino acid frequency in the YPS1009 proteome, using Fisher’s exact test and Benjamini-Hochberg FDR correction. Enrichments shown in Fig 3H were taken as the log2(fold difference) in frequency and shown only for statistically significant positions (FDR < 0.05, Fisher’s exact test). Quiescent aneuploids showed significant differences in amino acid composition at peaks detected in quiescence (Fig 3H); there were no significant differences for a comparable analysis done for log-phase cells (FDR > 0.05 in all cases). Data are available in GEO Accession #GSE269238. Custom scripts used are available in https://doi.org/10.5281/zenodo.17281997.

## Supporting information

S1 Fig Aneuploid and euploid yeast cells grow similarly during proliferative growth. **A)** HPLC analysis of glucose concentration in euploid and aneuploid strains at 8, 24, 72, 120 hours after the start of culturing. Euploid, YPS1009_Chr12, Chr14, and Chr15 were measured at 8 hours and beyond; other aneuploids were measured at 24 hours and beyond. All cultures showed <0.04 g/L glucose at 24 hours; some curves are superimposable in the figure. **B)** Representative brightfield images of live euploid and aneuploid cells during log-phase demonstrate that aneuploids do not show unusual morphologies during log phase. Scale bar, 25 µm. **C)** Representative Percoll density gradients. Nearly all euploid YPS1009 migrate in the dense fraction at day 4 of culturing, whereas YPS1009_Chr4 and _Chr12 aneuploids migrate in the light fraction indicative of non-dense cells. The data underlying this figure can be found in S2 Table.
(TIF)

S2 Fig Perturbing RQC genes affects quiescence phenotypes. **A)** Average and individual data points (*n* ≥ 3) of budded cells as shown in Fig 2 for indicated strains after 2 days of culturing. Asterisk, p < 0.05 comparing deletion mutants to WT; +, *p* < 0.1. *n* = 3, Fisher’s exact test applied to count data. **B)** Percent cells with morphology defects in YPS1009_Chr14 and _Chr15 after 2 days. Asterisk, *p* < 0.05 comparing strains with gene plasmids to EV or *rqc2Δ* to WT; *n* = 3, Fisher’s exact test. **C)** OD_600_ of euploid and aneuploid cultures harboring either empty vector (EV) or *RQC1* plasmid after 1 day of culturing. Asterisk, *p* < 0.05, paired *T* test, *n* = 3. **D)** Proportion of dense and light cells after 4 days. Asterisk, *p* < 0.05, Chi-squared test, *n* = 2. The data underlying this figure can be found in S2 Table.
(TIF)

S3 Fig Representative R12 stalling reporter expressed in denoted strains (columns) over time of culturing (rows) in denoted channels. Scale bar, 25 µm. See Fig 3 for stalling reporter details.
(TIF)

S4 Fig Ribosome profiling examples. **A)** Representative average read counts across all transcripts shows frame alignment across transcriptomes. **B)** Distribution of log_2_(fold difference) in normalized read counts (“Peak Heights”) for peaks scored as higher read count (normalized to gene body, see Methods) in aneuploids (orange) or in euploids (purple) in log phase (day 1) or quiescence (day 4) in two different replicates. In both replicates, a higher fraction of interrogated peaks were called significant during quiescence than log-phase and a higher fraction of those peaks had larger fold-differences in normalized read count. The data underlying this figure can be found in S2 Table. **C)** Representative traces at one transcript with significant aneuploid-enriched peak at the same position in both replicates (orange asterisk, FDR < 0.05 in both replicates).
(TIF)

S5 Fig Taxing RQC in euploid cells disrupts quiescence phenotypes. **A, B)** Proportion of dense and light cells after 4 days. Asterisk, *p* < 0.05, Chi-squared test, *n* = 2–4. The data underlying this figure can be found in S2 Table.
(TIF)

S6 Fig Cell cycle defects in aged aneuploid cells. **A)** Brightfield and DAPI images of euploid and aneuploid cells with notable morphology defects or nuclei polarity failures (white arrows). Scale bar, 5 µm. **B)** Average and individual data points of percent of budding cells lacking proper nuclear localization of Whi5-GFP at 2 days (*n* = 3). **C)** Representative brightfield and fluorescent images of budding Chr12 cells that lack nuclear Whi5-GFP at 2 days (red arrows). 95% of YPS1009_Chr12 cells that remained budded at 2 days were devoid of nuclear Whi5, consistent with cell-cycle entry that is dependent on Cln3-CDK activity. Scale bar, 5 µm. **D)** Average and individual data points (*n* = 3) of percent budding cells at 2 days in euploid cells harboring empty vector or *CLN3*, *CLN2*, or *CLB6* plasmids. Asterisk, *p* < 0.05, unpaired *T* test. Over-expression of these cyclins had only a minor effect on budding in euploid cells, but significantly exacerbated the budding defect in YPS1009_Chr12 or _Chr4 aneuploid cells. **E)** Anti-HA western blot of Cln3-6xHA tagged euploid and YPS1009_Chr12 strains during log-phase, 1 and 2 days after start of culturing. YPS1009_Chr12 cells accumulated Cln3 products, suggesting defects in Cln3 degradation, whereas euploid cells did not. The data underlying this figure can be found in S2 Table.
(TIF)

S7 Fig Aneuploids are not sensitive to MG132. Average and individual data points (*n* = 3) of **A)** relative growth rates or **B)** final OD_600_ of denoted strains (all lacking drug transporter *PDR5*) grown in 100 µM proteasome inhibitor MG132 relative to no-drug control. None of the aneuploids show increased sensitivity to MG132 compared to the euploid. **C)** Representative Ponceau S and anti-Ubiquitin western blot of euploid and denoted aneuploid strains grown in log phase or to day 2 of culturing when the arrest defect is apparent. Free ubiquitin runs at 8.6 kDa. See Methods for details. **D)** Maximum Hsp104-GFP signal per cell in wildtype and *rqc2∆* YPS1009_Chr4 and _Chr12 aneuploid cells. The *rqc2∆* aneuploids showed substantially less Hsp104-GFP signal (*, *p* < 3 × 10^−5^, Wilcoxon rank-sum test), confounding direct comparison to data shown in Fig 5 that quantifies the number of cells with foci. Nonetheless, cells lacking *RQC2* clearly show less signal compared to the paired euploid strain. The data underlying this figure can be found in S2 Table.
(TIF)

S8 Fig Representative Hsp104-GFP foci in denoted strains (rows) with specified plasmids (columns). Quiescent cells are known to express some nuclear Hsp104-GFP at this time point. Small puncta that did not overlap nuclear signal (white arrows) were scored as foci; nuclear Hsp104-GFP that overlapped with DAPI signal (red arrows) was not scored. Fig 5 quantified the number of cells with one or more foci as outlined in the legend and Methods. Scale bar, 25 µm.
(TIF)

S1 Data Excel file containing replicate-averaged log_2_(fold change) in barcode abundance for the genetic screen described in Fig 2. (XLSX)

S2 Data Excel file containing the replicate-averaged log_2_(fold difference) in normalized transcript abundance for the 963 genes displayed in Fig 1D. (XLSX)

S1 Table Strain list. Excel file containing yeast strain information for each strain used in this study.
(XLSX)

S2 Table Supporting data. Excel file containing all numerical values for data underlying displayed summary data. Data for each figure is indicated by the name of each sheet.
(XLSX)

S1 Raw Images PDF file containing raw images of all the western blots presented or quantified in the paper. (PDF)

## Abbreviations

DS: Down syndrome
DUB: deubiquitinase
FDR: false discovery rate
NTC: nourseothricin
RQC: Ribosome Quality Control
