# Supplementary figures and images for: Chromosome duplication causes premature aging via defects in ribosome quality control
Source: PLoS Biol. 2025 Nov 17;23(11):e3003509. doi: 10.1371/journal.pbio.3003509 (PMC12633879; doi:10.1371/journal.pbio.3003509)

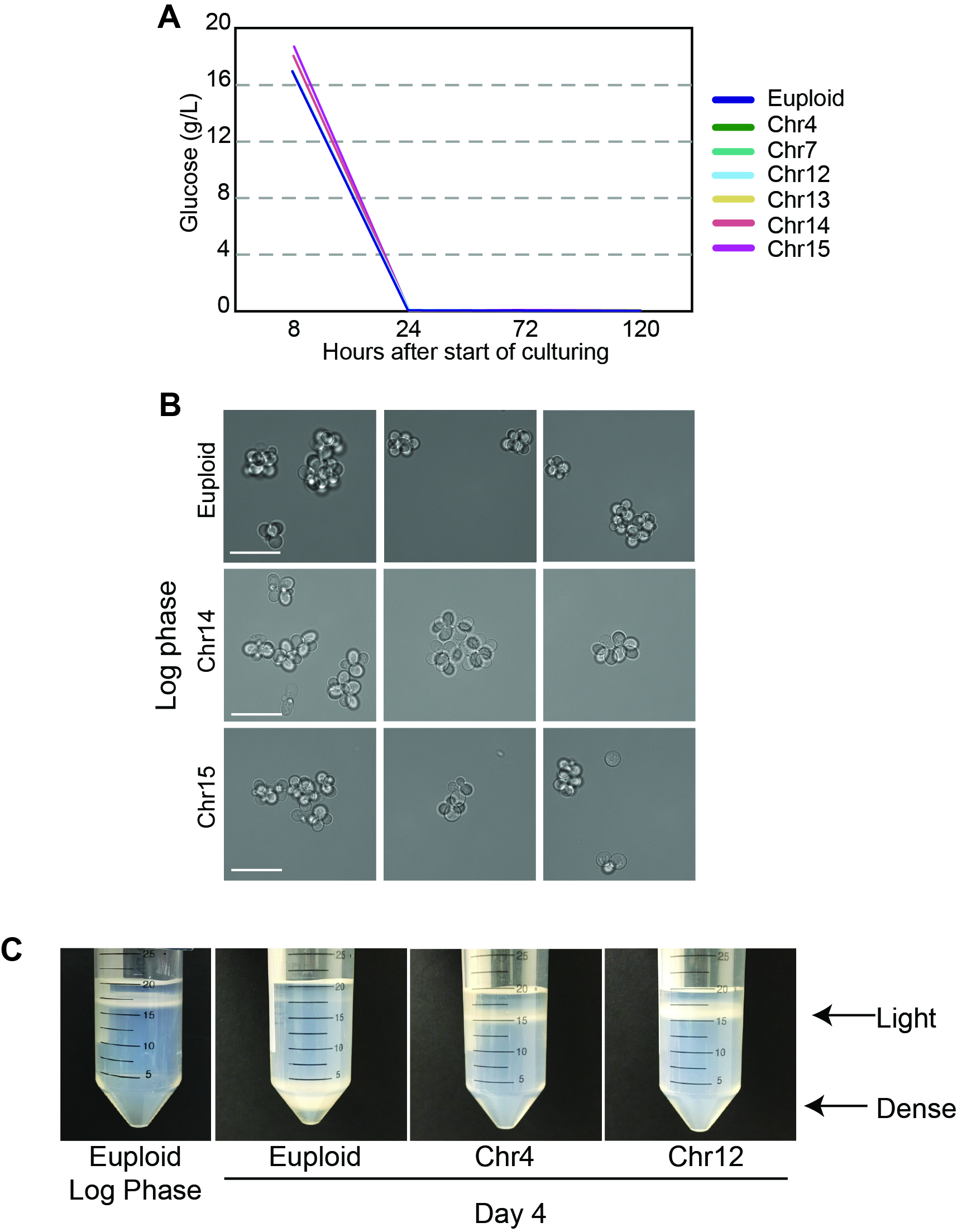

Supplement: S1 Fig — A) HPLC analysis of glucose concentration in euploid and aneuploid strains at 8, 24, 72, 120 hours after the start of culturing. Euploid, YPS1009_Chr12, Chr14, and Chr15 were measured at 8 hours and beyond; other aneuploids were measured at 24 hours and beyond. All cultures showed <0.04 g/L glucose at 24 hours; some curves are superimposable in the figure. B) Representative brightfield images of live euploid and aneuploid cells during log-phase demonstrate that aneuploids do not show unusual morphologies during log phase. Scale bar, 25 µm. C) Representative Percoll density gradients. Nearly all euploid YPS1009 migrate in the dense fraction at day 4 of culturing, whereas YPS1009_Chr4 and _Chr12 aneuploids migrate in the light fraction indicative of non-dense cells. The data underlying this figure can be found in S2 Table. (TIF) [file pbio.3003509.s001.tif]

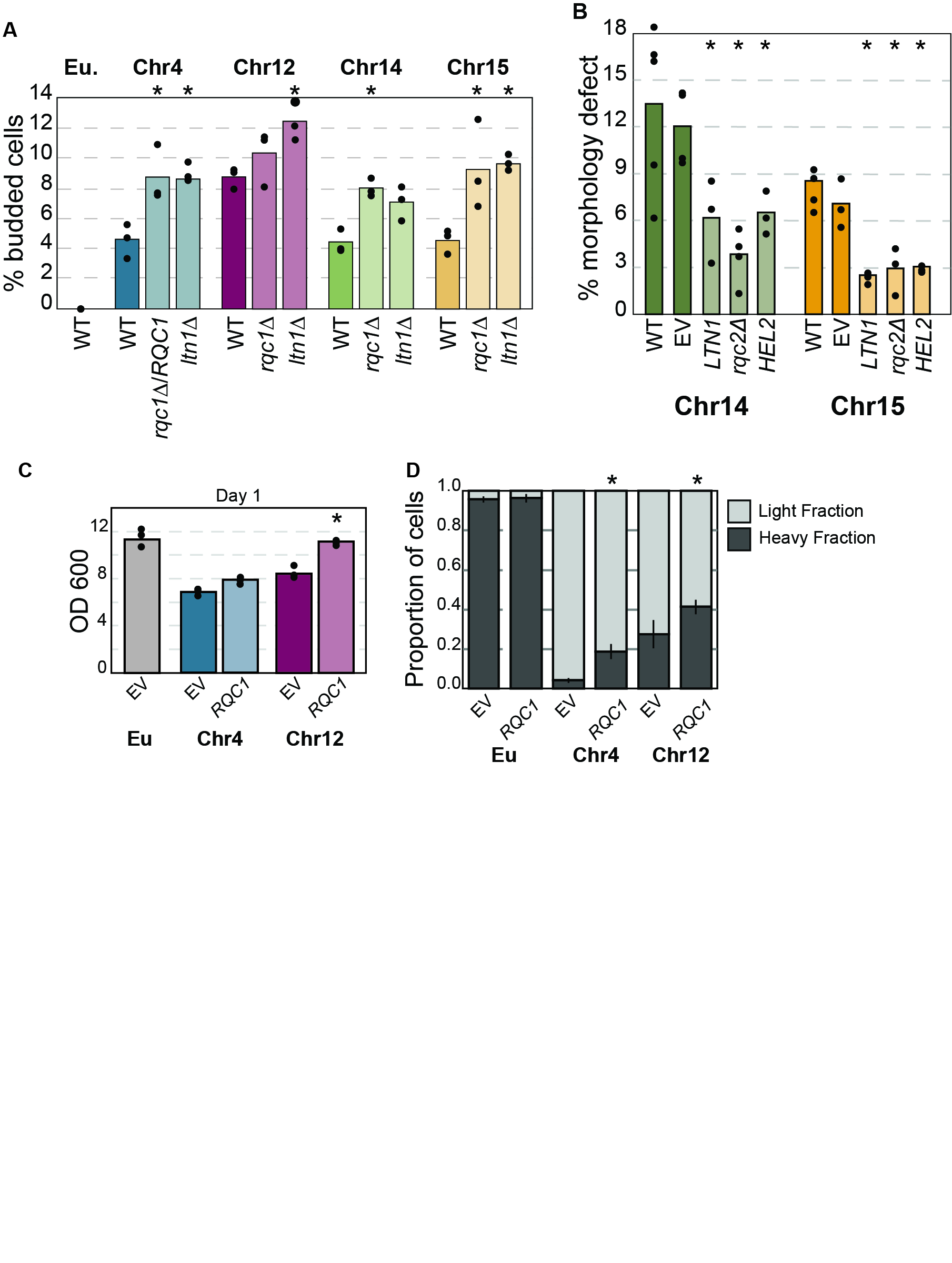

Supplement: S2 Fig — A) Average and individual data points (n ≥ 3) of budded cells as shown in Fig 2 for indicated strains after 2 days of culturing. Asterisk, p < 0.05 comparing deletion mutants to WT; +, p < 0.1. n = 3, Fisher’s exact test applied to count data. B) Percent cells with morphology defects in YPS1009_Chr14 and _Chr15 after 2 days. Asterisk, p < 0.05 comparing strains with gene plasmids to EV or rqc2Δ to WT; n = 3, Fisher’s exact test. C) OD600 of euploid and aneuploid cultures harboring either empty vector (EV) or RQC1 plasmid after 1 day of culturing. Asterisk, p < 0.05, paired T test, n = 3. D) Proportion of dense and light cells after 4 days. Asterisk, p < 0.05, Chi-squared test, n = 2. The data underlying this figure can be found in S2 Table. (TIF) [file pbio.3003509.s002.tif]

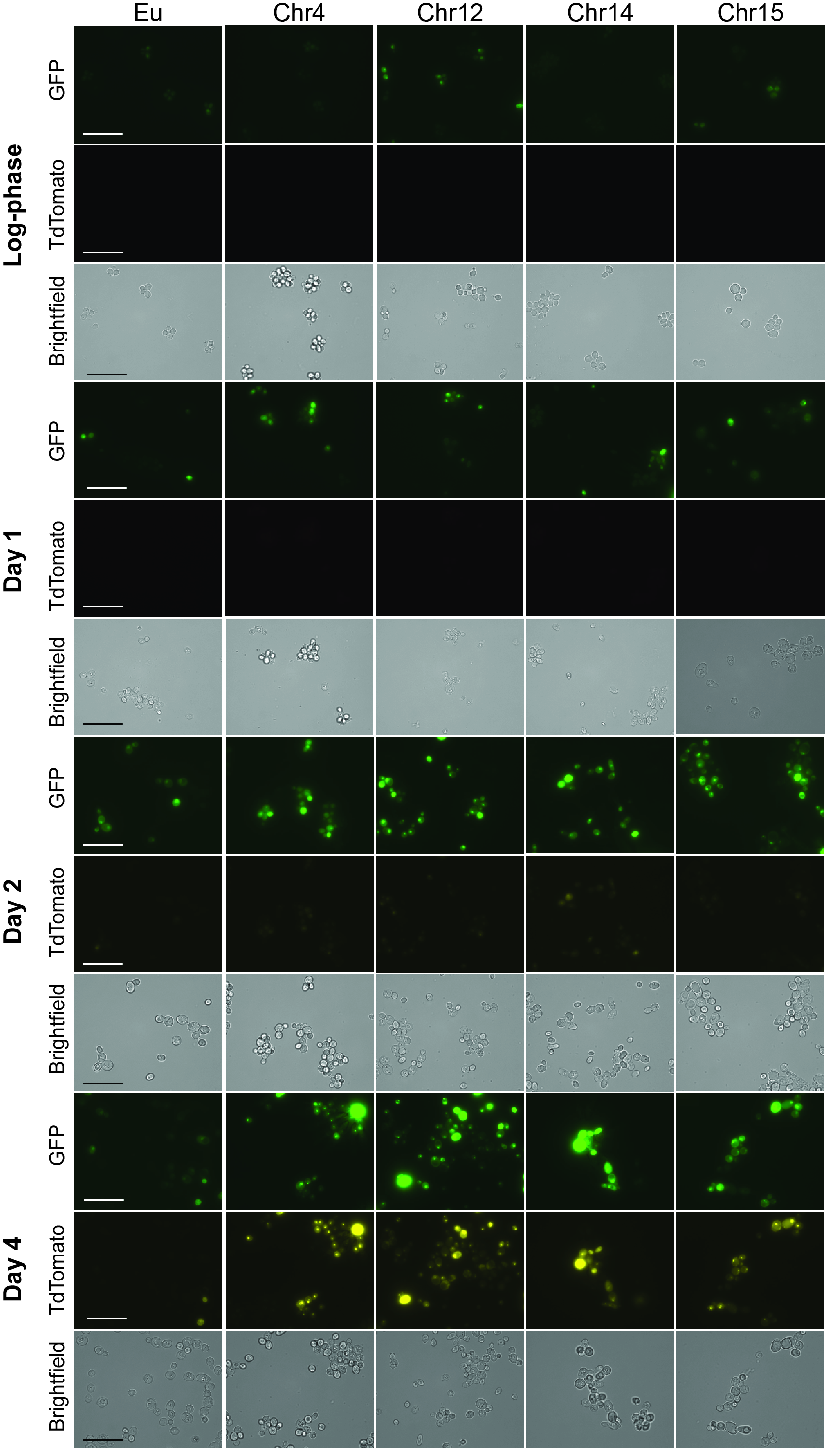

Supplement: S3 Fig — Scale bar, 25 µm. See Fig 3 for stalling reporter details. (TIF) [file pbio.3003509.s003.tif]

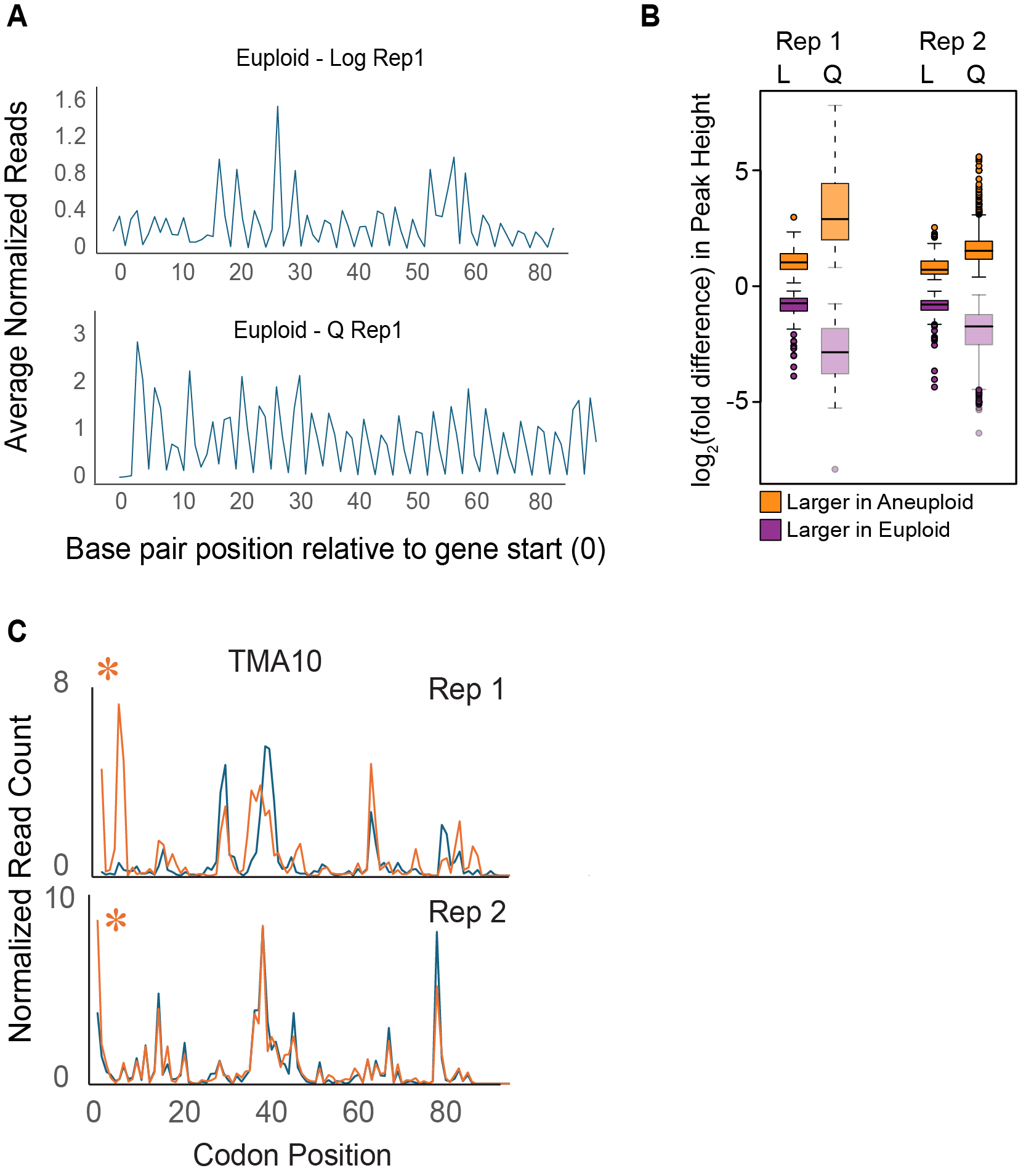

Supplement: S4 Fig — A) Representative average read counts across all transcripts shows frame alignment across transcriptomes. B) Distribution of log2(fold difference) in normalized read counts (“Peak Heights”) for peaks scored as higher read count (normalized to gene body, see Methods) in aneuploids (orange) or in euploids (purple) in log phase (day 1) or quiescence (day 4) in two different replicates. In both replicates, a higher fraction of interrogated peaks were called significant during quiescence than log-phase and a higher fraction of those peaks had larger fold-differences in normalized read count. The data underlying this figure can be found in S2 Table. C) Representative traces at one transcript with significant aneuploid-enriched peak at the same position in both replicates (orange asterisk, FDR < 0.05 in both replicates). (TIF) [file pbio.3003509.s004.tif]

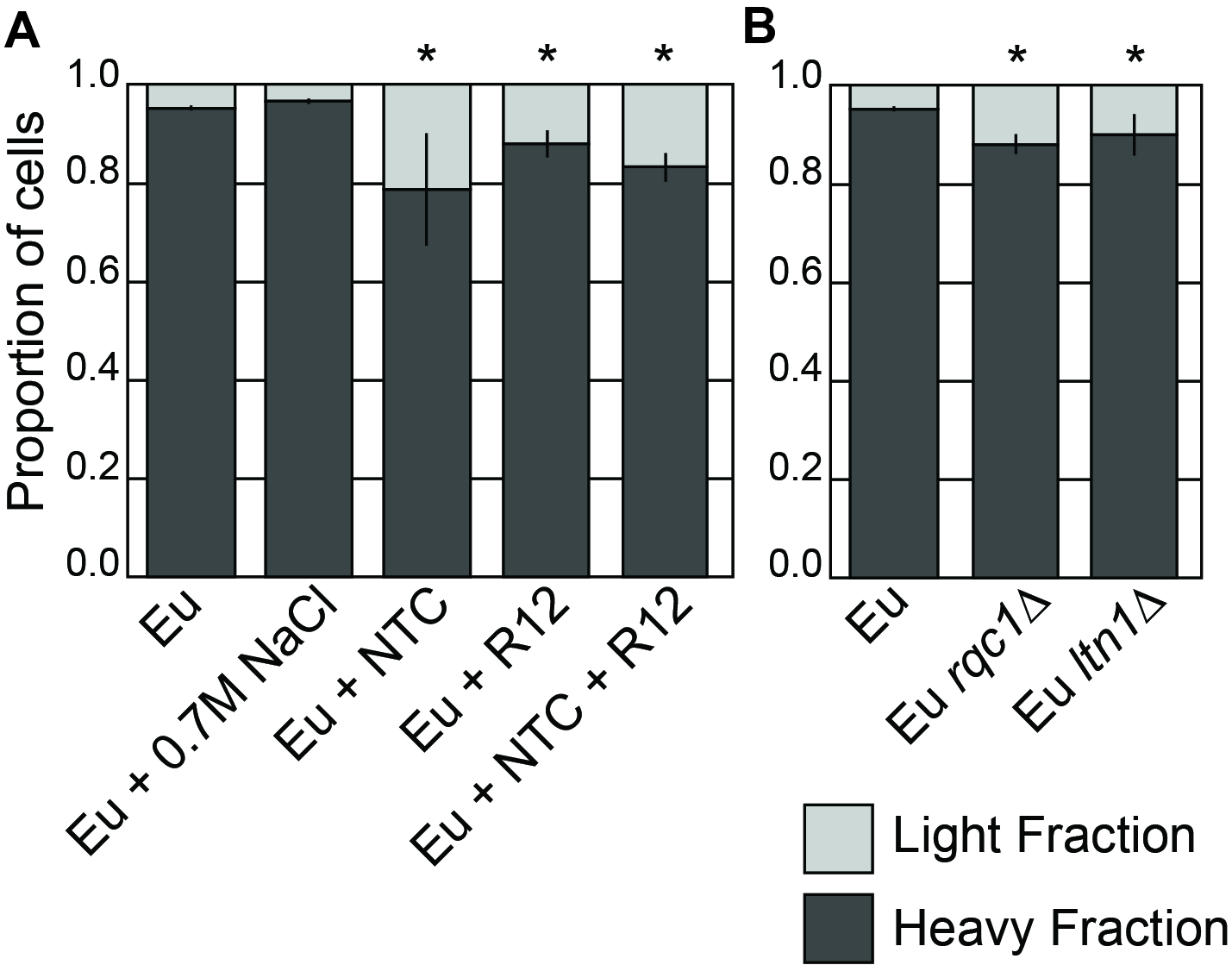

Supplement: S5 Fig — A, B) Proportion of dense and light cells after 4 days. Asterisk, p < 0.05, Chi-squared test, n = 2–4. The data underlying this figure can be found in S2 Table. (TIF) [file pbio.3003509.s005.tif]

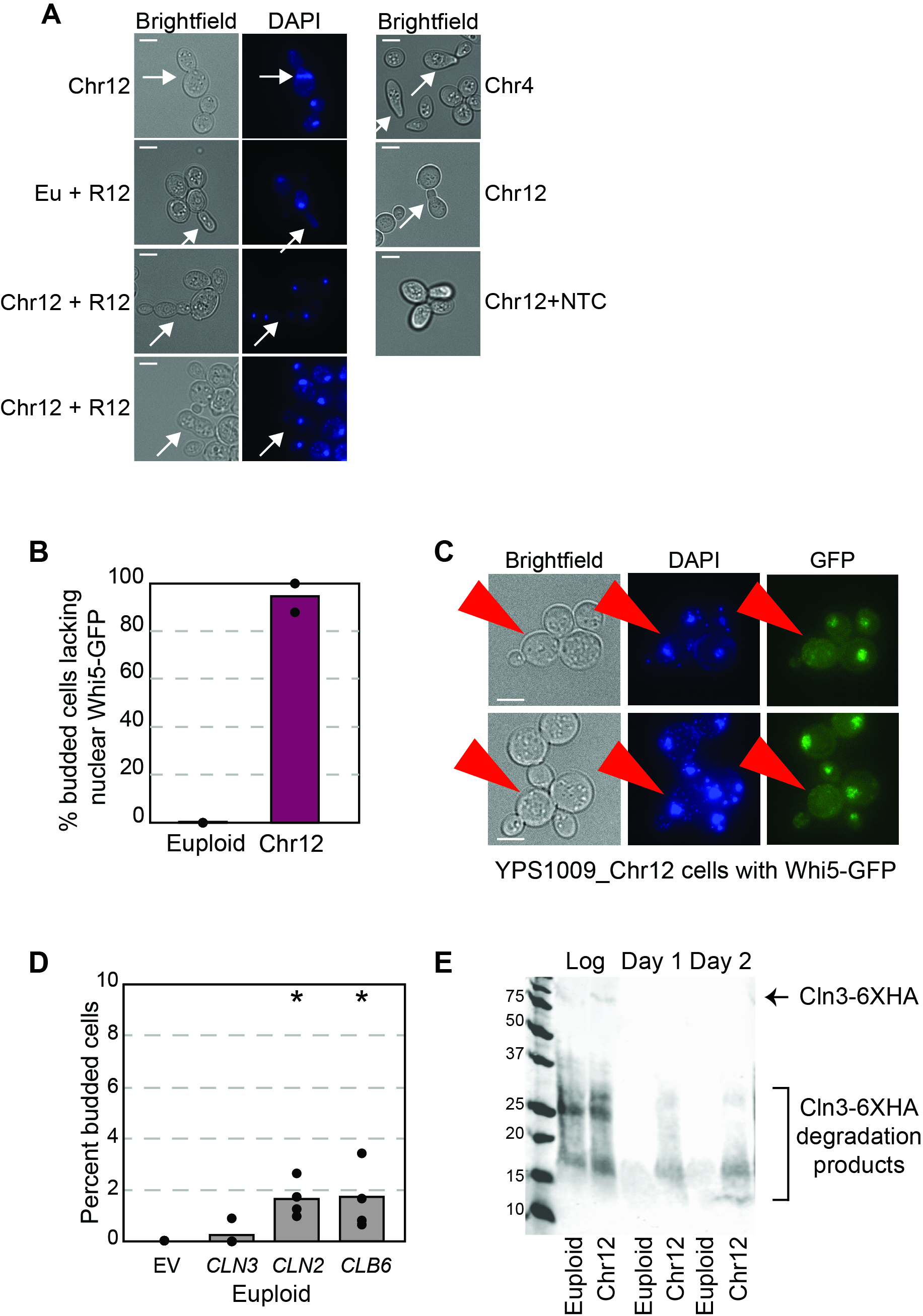

Supplement: S6 Fig — A) Brightfield and DAPI images of euploid and aneuploid cells with notable morphology defects or nuclei polarity failures (white arrows). Scale bar, 5 µm. B) Average and individual data points of percent of budding cells lacking proper nuclear localization of Whi5-GFP at 2 days (n = 3). C) Representative brightfield and fluorescent images of budding Chr12 cells that lack nuclear Whi5-GFP at 2 days (red arrows). 95% of YPS1009_Chr12 cells that remained budded at 2 days were devoid of nuclear Whi5, consistent with cell-cycle entry that is dependent on Cln3-CDK activity. Scale bar, 5 µm. D) Average and individual data points (n = 3) of percent budding cells at 2 days in euploid cells harboring empty vector or CLN3, CLN2, or CLB6 plasmids. Asterisk, p < 0.05, unpaired T test. Over-expression of these cyclins had only a minor effect on budding in euploid cells, but significantly exacerbated the budding defect in YPS1009_Chr12 or _Chr4 aneuploid cells. E) Anti-HA western blot of Cln3-6xHA tagged euploid and YPS1009_Chr12 strains during log-phase, 1 and 2 days after start of culturing. YPS1009_Chr12 cells accumulated Cln3 products, suggesting defects in Cln3 degradation, whereas euploid cells did not. The data underlying this figure can be found in S2 Table. (TIF) [file pbio.3003509.s006.tif]

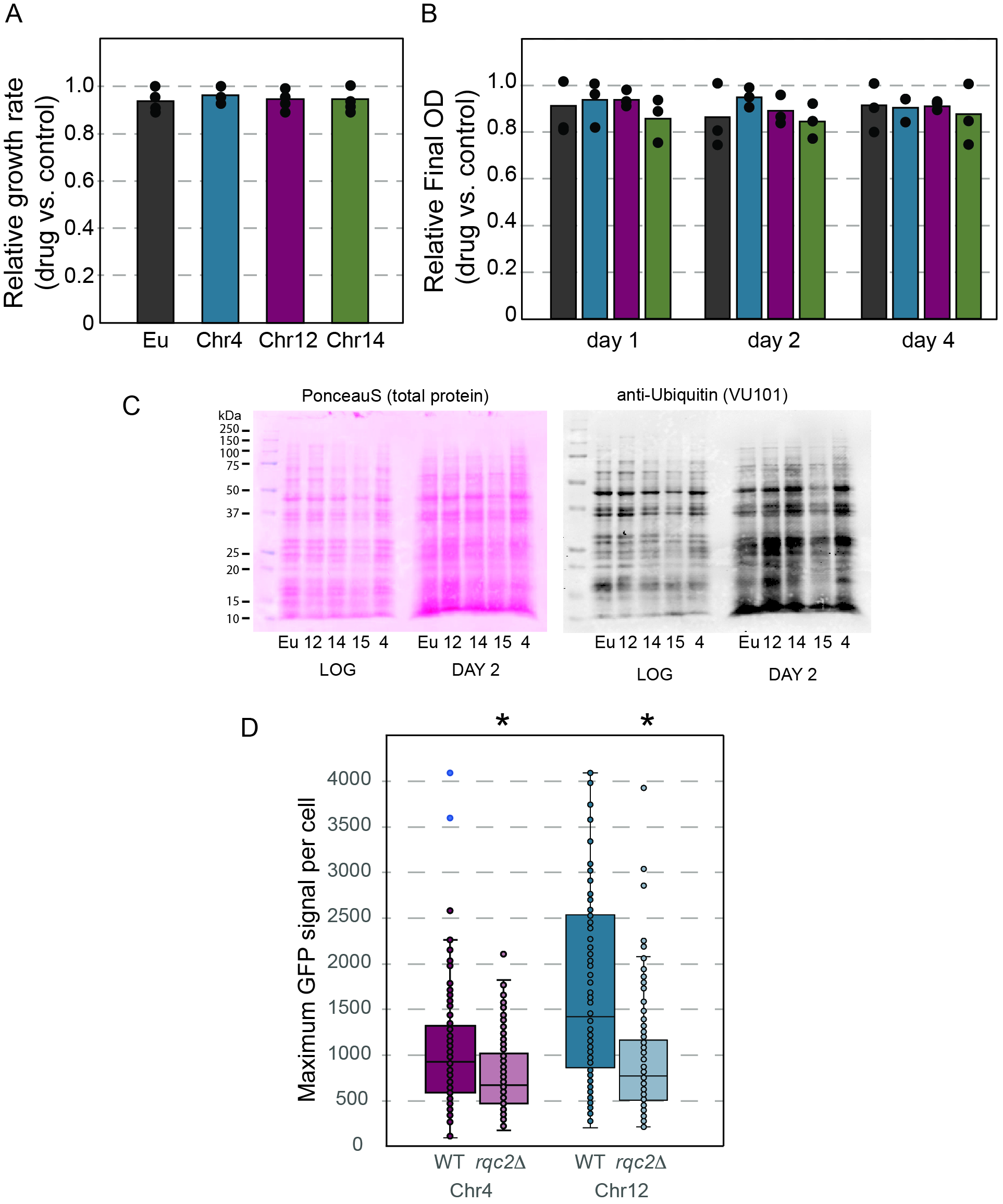

Supplement: S7 Fig — Average and individual data points (n = 3) of A) relative growth rates or B) final OD600 of denoted strains (all lacking drug transporter PDR5) grown in 100 µM proteasome inhibitor MG132 relative to no-drug control. None of the aneuploids show increased sensitivity to MG132 compared to the euploid. C) Representative Ponceau S and anti-Ubiquitin western blot of euploid and denoted aneuploid strains grown in log phase or to day 2 of culturing when the arrest defect is apparent. Free ubiquitin runs at 8.6 kDa. See Methods for details. D) Maximum Hsp104-GFP signal per cell in wildtype and rqc2∆ YPS1009_Chr4 and _Chr12 aneuploid cells. The rqc2∆ aneuploids showed substantially less Hsp104-GFP signal (*, p < 3 × 10^−5^, Wilcoxon rank-sum test), confounding direct comparison to data shown in Fig 5 that quantifies the number of cells with foci. Nonetheless, cells lacking RQC2 clearly show less signal compared to the paired euploid strain. The data underlying this figure can be found in S2 Table. (TIF) [file pbio.3003509.s007.tif]

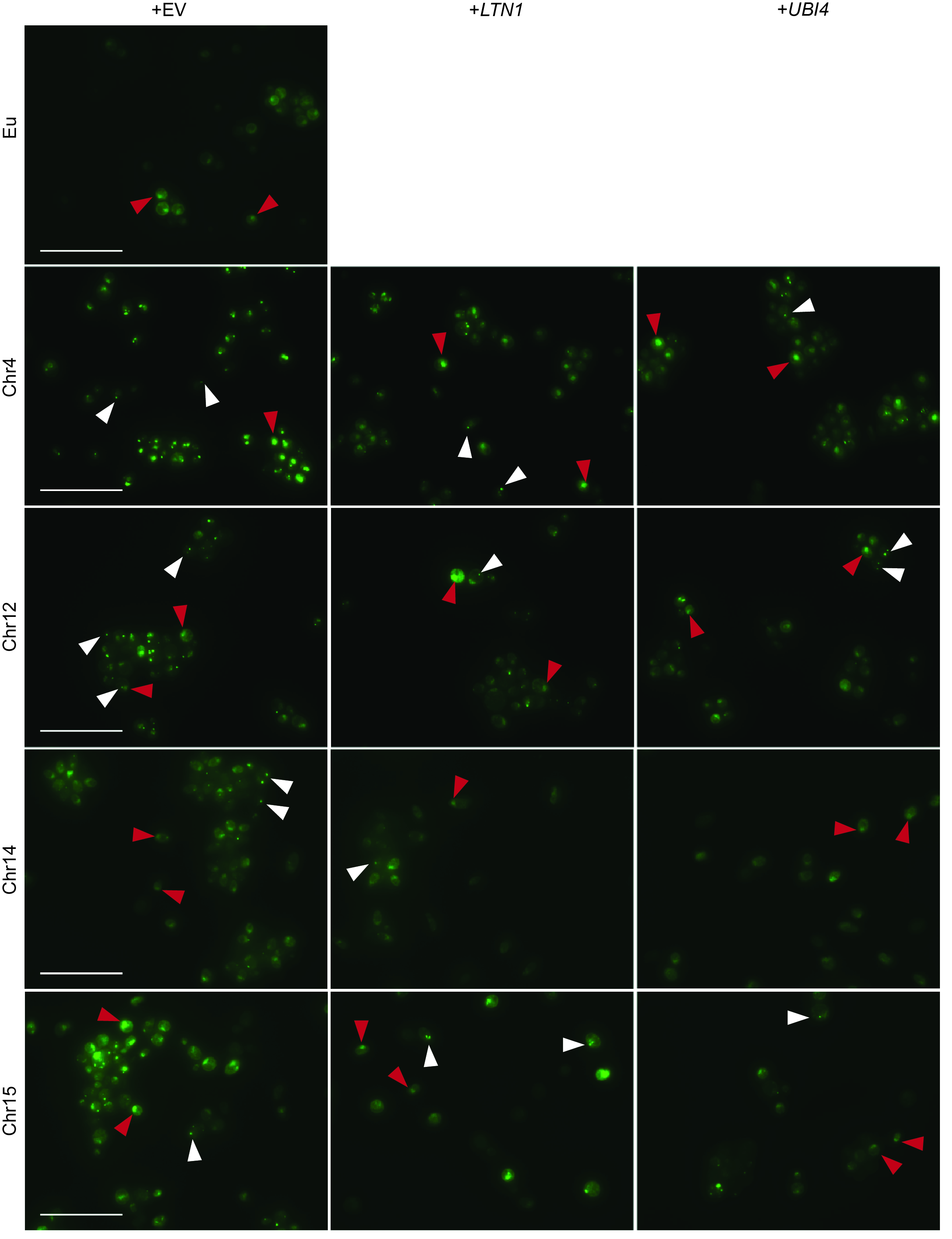

Supplement: S8 Fig — Quiescent cells are known to express some nuclear Hsp104-GFP at this time point. Small puncta that did not overlap nuclear signal (white arrows) were scored as foci; nuclear Hsp104-GFP that overlapped with DAPI signal (red arrows) was not scored. Fig 5 quantified the number of cells with one or more foci as outlined in the legend and Methods. Scale bar, 25 µm. (TIF) [file pbio.3003509.s008.tif]
