# Supplementary material for: Chromosome duplication causes premature aging via defects in ribosome quality control
Source: PLoS Biol. 2025 Nov 17;23(11):e3003509. doi: 10.1371/journal.pbio.3003509 (PMC12633879; doi:10.1371/journal.pbio.3003509)
Supplement: S1 Raw Images — (PDF) [file pbio.3003509.s013.pdf]

Fig 3B

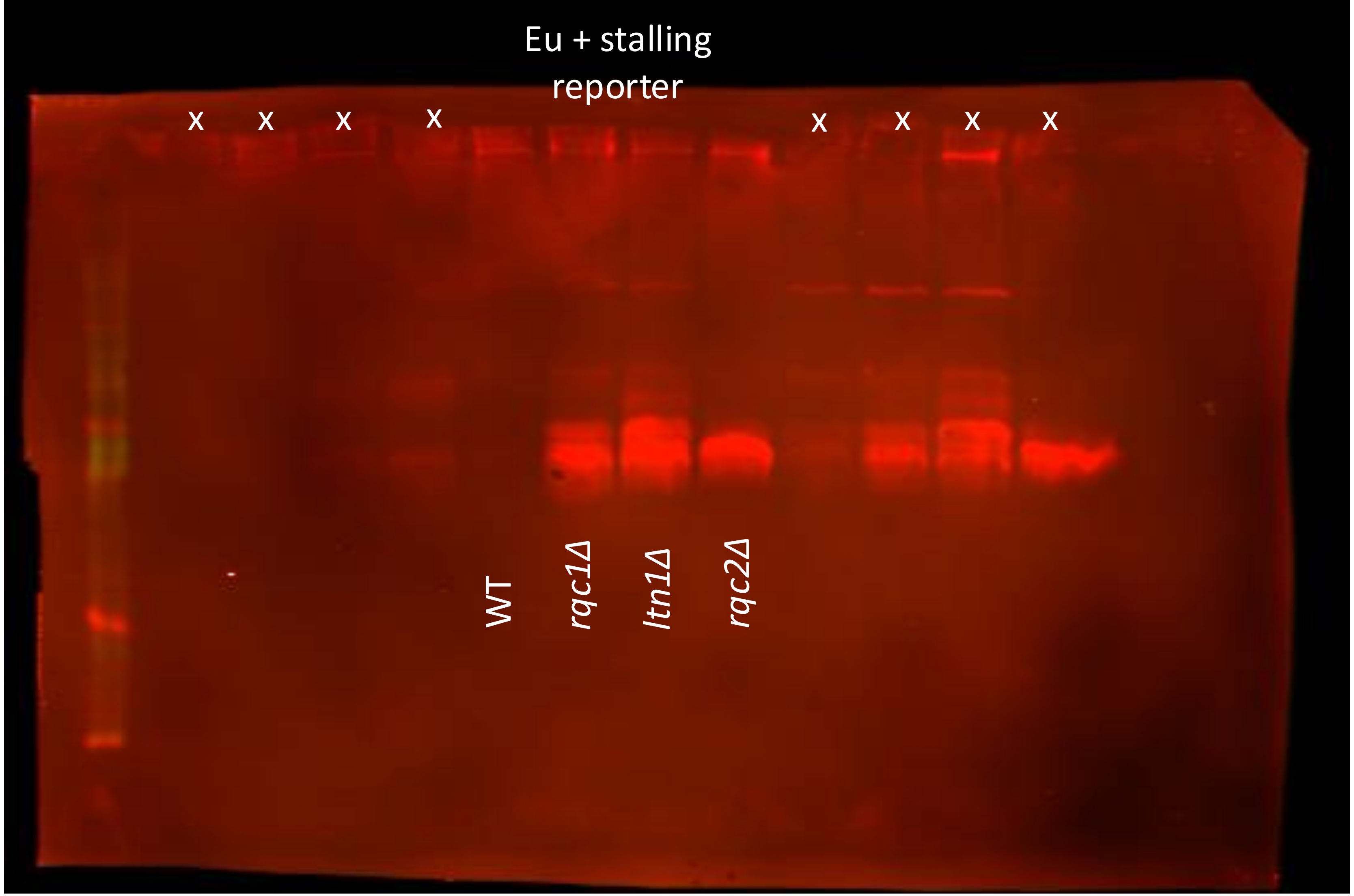

Acquired on LICOR Odyssey imager

Figure 3C (western blots underlying the quantification in Fig3C)

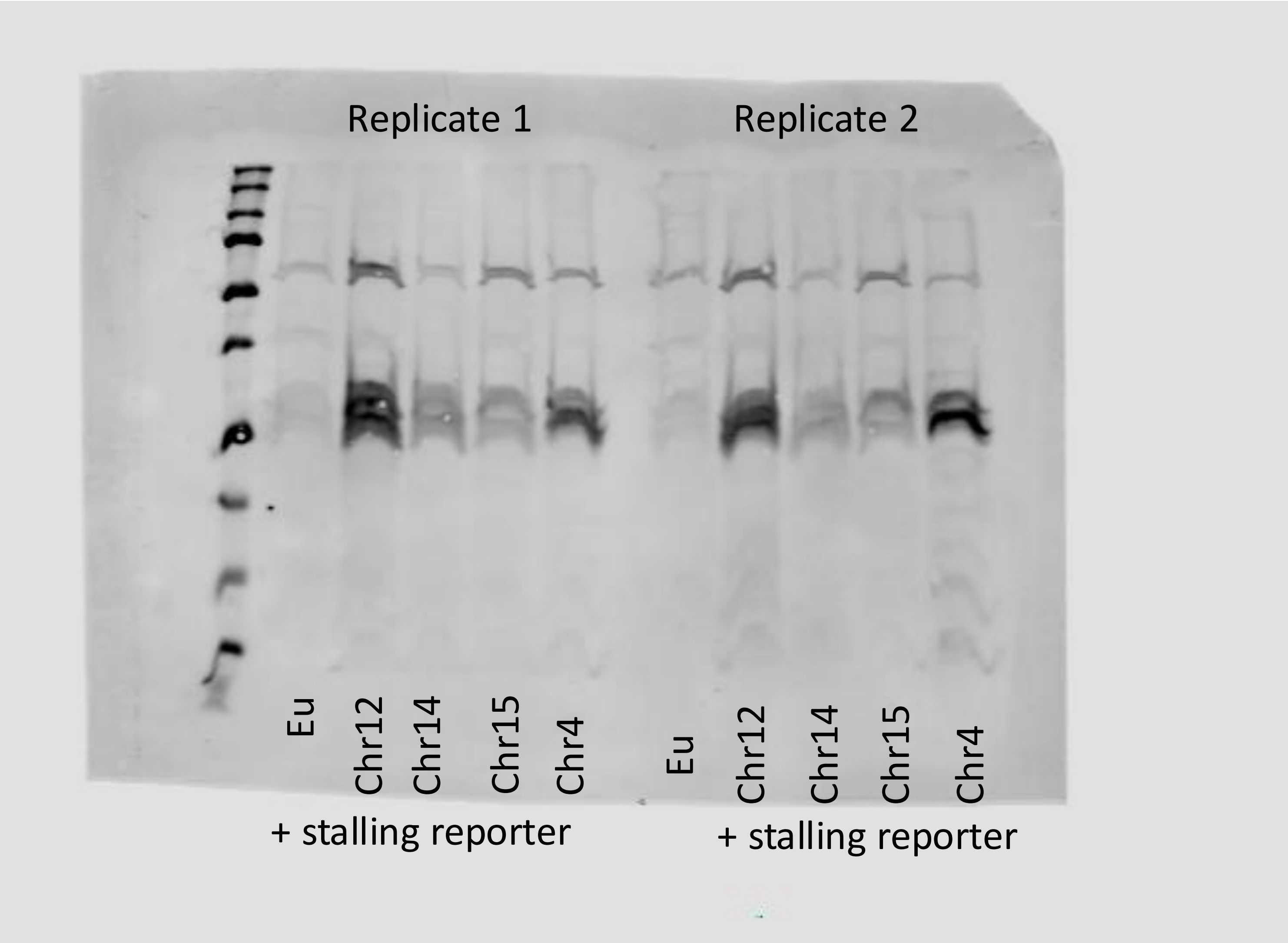

Acquired on LICOR Odyssey imager

Figure 3B-C (western blots underlying the quantification in Fig3C)

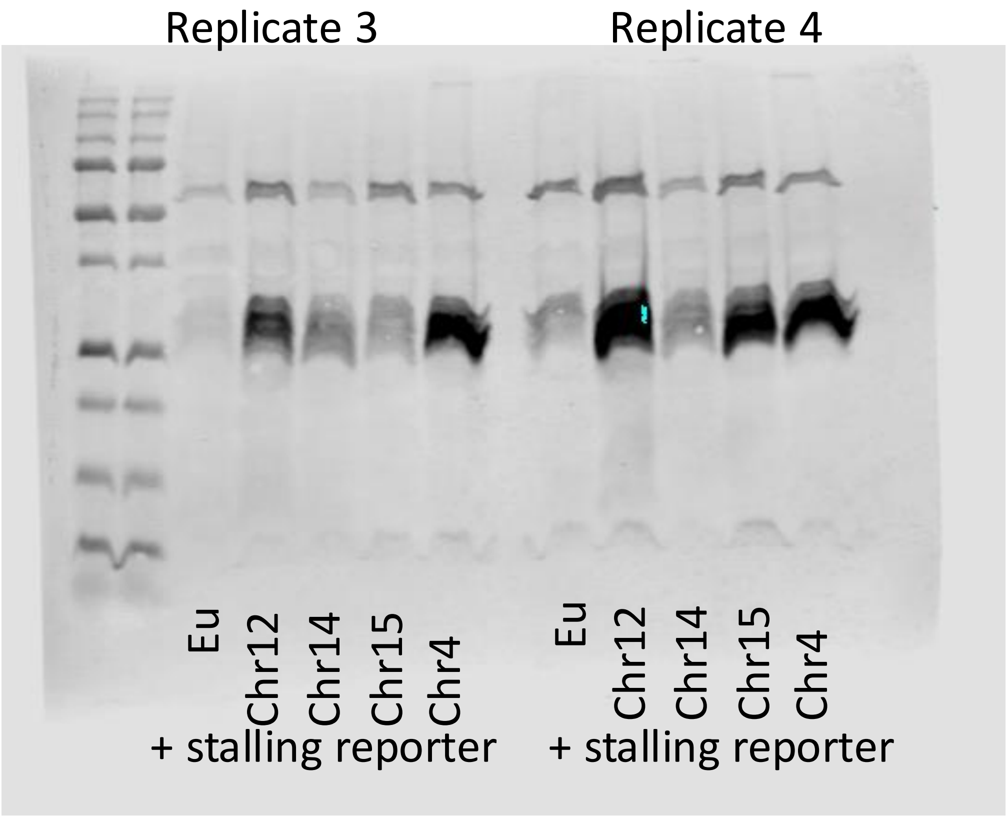

Acquired on LICOR Odyssey imager

Figure 4A

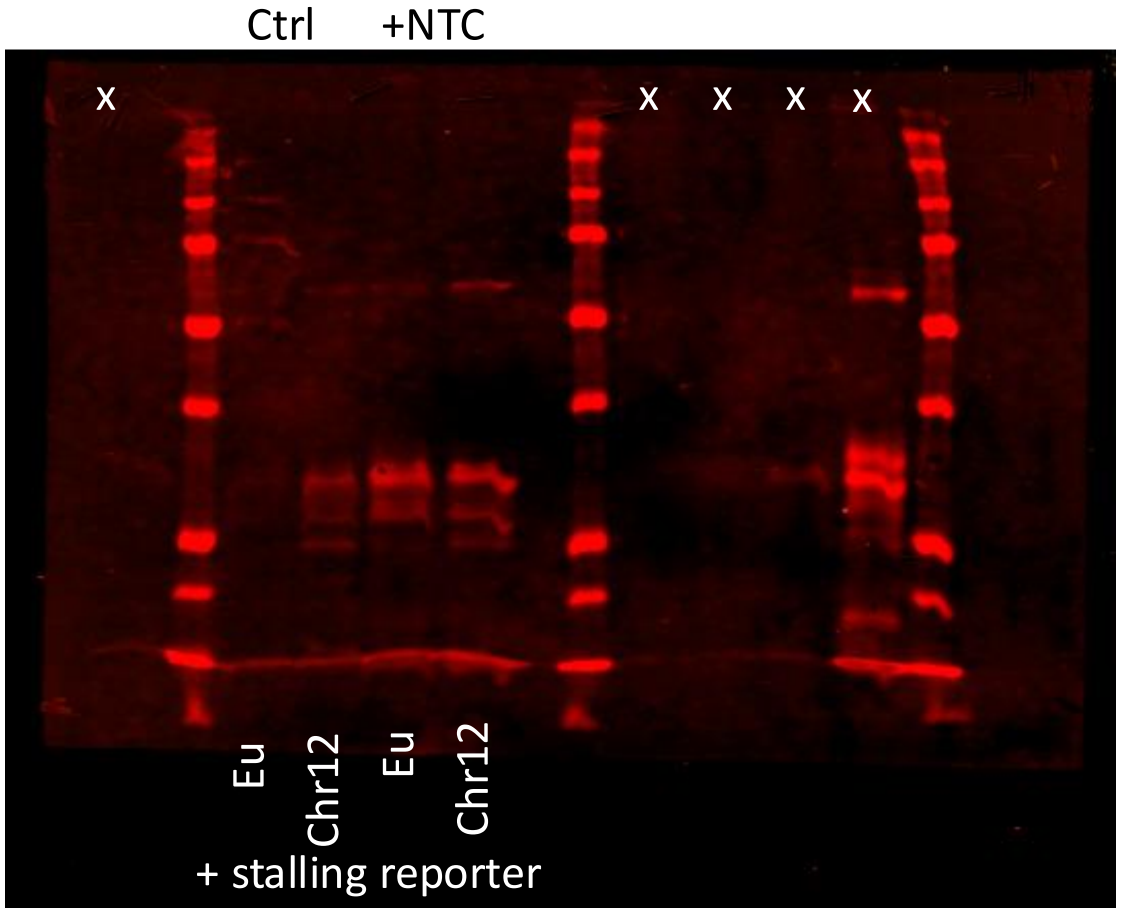

Acquired on LICOR Odyssey imager

Figure S6E

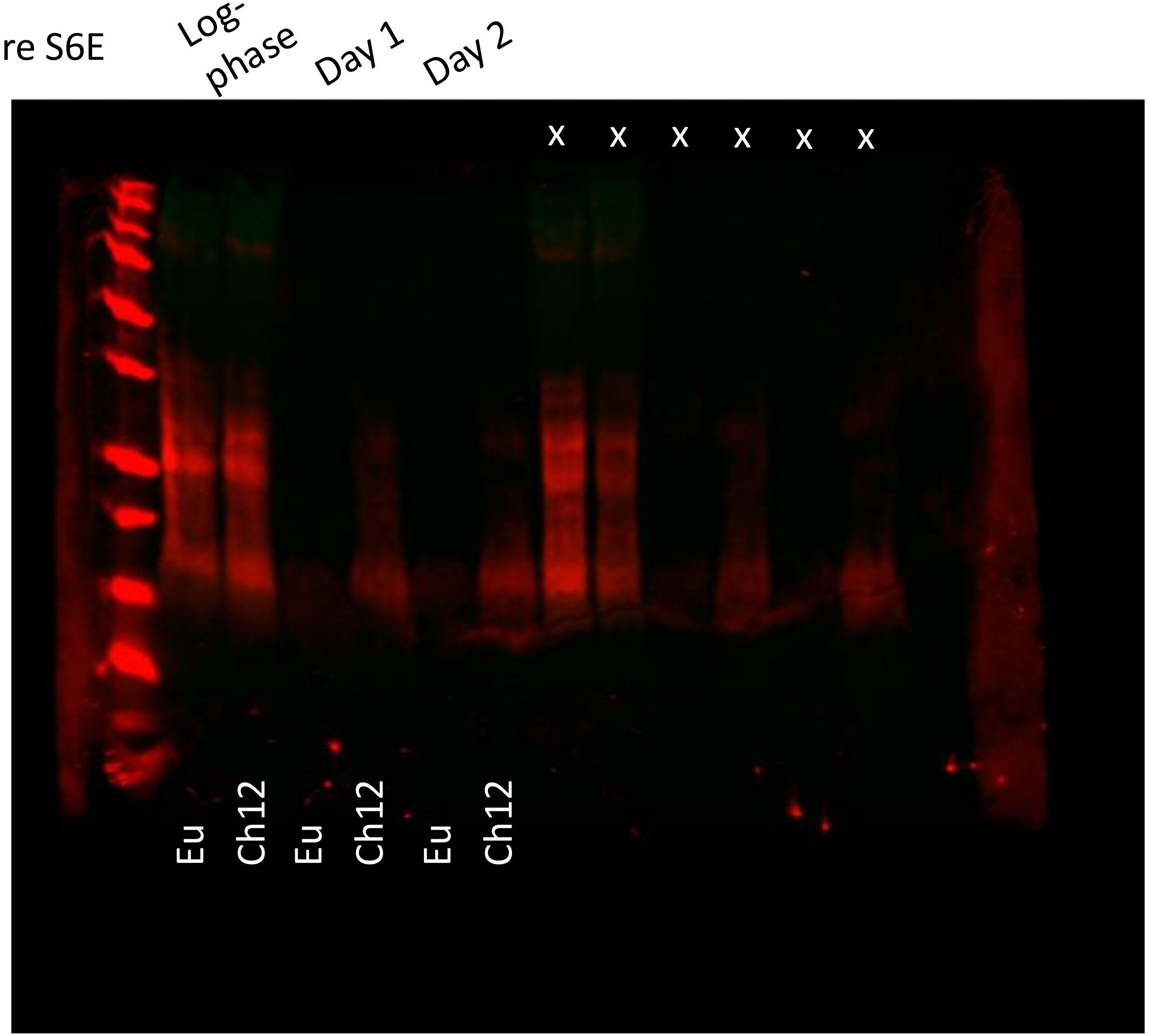

Acquired on LICOR Odyssey imager

Figure S7C

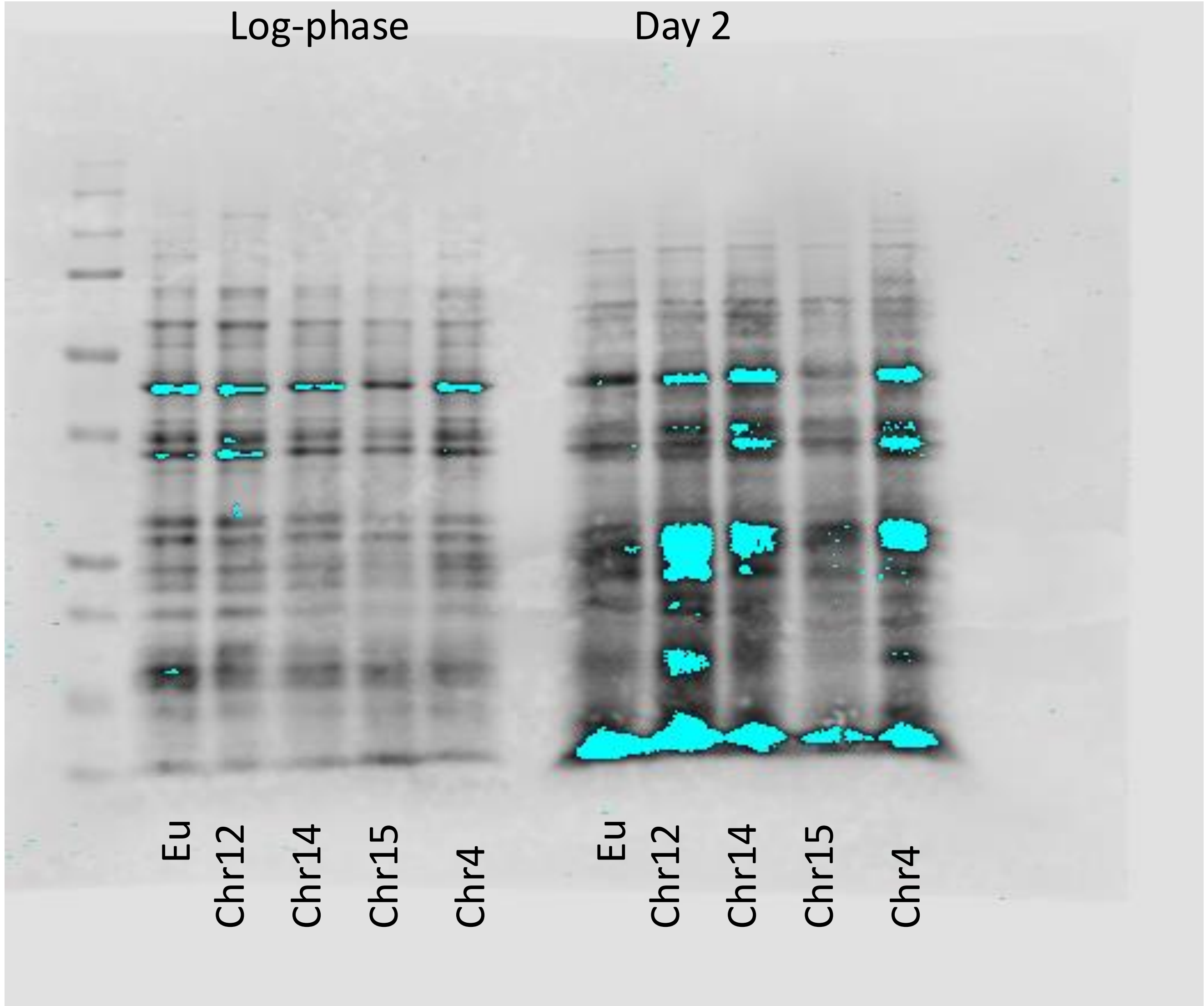

Acquired on LICOR Odyssey imager
